# Impaired activation of plasmacytoid dendritic cells *via* toll-like receptor 7/9 and STING is mediated by melanoma-derived immunosuppressive cytokines and metabolic drift

**DOI:** 10.3389/fimmu.2023.1227648

**Published:** 2024-01-03

**Authors:** Matilde Monti, Giorgia Ferrari, Valentina Grosso, Francesco Missale, Mattia Bugatti, Valeria Cancila, Stefania Zini, Agnese Segala, Luca La Via, Francesca Consoli, Matteo Orlandi, Alessandra Valerio, Claudio Tripodo, Marzia Rossato, William Vermi

**Affiliations:** ^1^ Department of Molecular and Translational Medicine, University of Brescia, Brescia, Italy; ^2^ Department of Biotechnology, University of Verona, Verona, Italy; ^3^ Department of Head & Neck Oncology & Surgery Otorhinolaryngology, Nederlands Kanker Instituut, Amsterdam, Netherlands; ^4^ Tumor Immunology Unit, Department of Health Sciences, University of Palermo, Palermo, Italy; ^5^ Oncology Unit, Azienda Socio Sanitaria Territoriale (ASST) Spedali Civili di Brescia, Brescia, Italy; ^6^ IFOM ETS, the AIRC Institute of Molecular Oncology, Milan, Italy; ^7^ Department of Pathology and Immunology, Washington University School of Medicine, Saint Louis, MO, United States

**Keywords:** melanoma, plasmacytoid dendritic cells, toll-like receptor, interferon, cGAS-STING, tumor microenvironment, glycolysis, TGF-β

## Abstract

**Introduction:**

Plasmacytoid dendritic cells (pDCs) infiltrate a large set of human cancers. Interferon alpha (IFN-α) produced by pDCs induces growth arrest and apoptosis in tumor cells and modulates innate and adaptive immune cells involved in anti-cancer immunity. Moreover, effector molecules exert tumor cell killing. However, the activation state and clinical relevance of pDCs infiltration in cancer is still largely controversial. In Primary Cutaneous Melanoma (PCM), pDCs density decreases over disease progression and collapses in metastatic melanoma (MM). Moreover, the residual circulating pDC compartment is defective in IFN-α production.

**Methods:**

The activation of tumor-associated pDCs was evaluated by *in silico* and microscopic analysis. The expression of human myxovirus resistant protein 1 (MxA), as surrogate of IFN-α production, and proximity ligation assay (PLA) to test dsDNA-cGAS activation were performed on human melanoma biopsies. Moreover, IFN-α and CXCL10 production by *in vitro* stimulated (i.e. with R848, CpG-A, ADU-S100) pDCs exposed to melanoma cell lines supernatants (SN-mel) was tested by intracellular flow cytometry and ELISA. We also performed a bulk RNA-sequencing on SN-mel-exposed pDCs, resting or stimulated with R848. Glycolytic rate assay was performed on SN-mel-exposed pDCs using the Seahorse XFe24 Extracellular Flux Analyzer.

**Results:**

Based on a set of microscopic, functional and *in silico* analyses, we demonstrated that the melanoma milieu directly impairs IFN-α and CXCL10 production by pDCs *via* TLR-7/9 and cGAS-STING signaling pathways. Melanoma-derived immunosuppressive cytokines and a metabolic drift represent relevant mechanisms enforcing pDC-mediated melanoma escape.

**Discussion:**

These findings propose a new window of intervention for novel immunotherapy approaches to amplify the antitumor innate immune response in cutaneous melanoma (CM).

## Introduction

The clinical success of immune checkpoint blockades (ICBs) in patients with melanoma has driven the therapeutic revolution in cancer approach and new combinations of ICBs have significantly improved the outcome of patients with metastatic melanoma (MM) (1, 2). More recently, advances with ICBs have led to the approval of anti-PD1 agents and Pembrolizumab in the adjuvant setting, resulting in a higher recurrence-free survival in comparison to placebo (3–7). Unfortunately, half of the surgically resected patients relapses and do not show long-lasting benefit with ICBs (2), suggesting the need for a better characterization of predictive biomarkers for patient selection and for novel strategies to overcome resistance.

Features of the immune contexture of cancer predict prognosis and response to ICBs. Among immune cells involved in anti-tumor immune response, Plasmacytoid Dendritic cells (pDCs) play a crucial role bridging the innate and adaptive immunity (8). pDCs are the major type I interferon (I-IFN) producing cells upon nucleic acids sensing through Toll-like receptor (TLR) 7- and 9-dependent signaling pathways (9, 10). In addition, pDCs express other Pattern Recognition Receptors (PRR), such as C-type lectin receptors (CLRs), RIG-I-like receptors (RLRs), NOD-like receptors (NLRs), and cyclic guanosine monophosphate-adenosine monophosphate synthase (cGAS) (11). Innate immune response is also promoted by free endogenous DNA (12) and pDCs are able to sense cytosolic DNA through cGAS–STING (stimulator of interferon genes) pathway eliciting a potent I-IFN production independently of TLR7/9 (13–15). interferon-α (IFN-α) production directly affects tumor growth by inducing cell-cycle arrest and apoptosis of cancer cells and inhibiting angiogenesis (16, 17). Moreover, IFN-α modulates innate and adaptive immune cells involved in anti-cancer immunity (18–20), by inducing the paracrine production of pro-inflammatory chemokines (i.e. CXCL9, CXCL10, CXCL11) (21) and driving TH1 polarization of immune cells (22). In the last years, pDCs have been identified among the restricted cell subsets expressing the IFN-λ functional receptor (IFNλR) other than producers of type III interferons (IFN-λ or IL-28/IL-29), which play potent anti-viral activities and trigger IFN-α production during tumor progression (23). Nucleic acid-sensing mechanisms are druggable and, in combination with radiation therapy or chemotherapy, they boost the anti-tumor immune response by triggering DNA damage-induced immunogenic cell death and innate immune activation (24–27).

A large set of studies (28–30) have documented that pDCs recruited to the tumor microenvironment (TME) often display a non-activated state. Tumor cells and cells of the microenvironment produce immunosuppressive cytokines (e.g., PGE2, IL-10, and TGF-β), oncometabolites (e.g., lactic acid) and express ligands of IFN inhibitory receptors (e.g., BST2) that hijack IFN-α production by pDCs (28, 31). Furthermore, pDCs execute a tolerogenic activity in the TME by inducing regulatory T cells (Tregs) (32). This is mediated by the expression of indoleamine 2,3-dioxygenase (IDO), inducible T cell co-stimulator ligand (ICOSL), tumor necrosis factor ligand superfamily member 4 (TNFSF4; also known as OX40L) or programmed death-ligand 1 (PD-L1) (33–36). Therefore, tumor-associated pDCs (TA-pDCs) maintain immunosuppression and their density can predict poor outcome in breast cancer, ovarian cancer and melanoma (37).

Preclinical data suggest that the activation of TA-pDCs by TLR-7/9 agonists administration amplifies the local and systemic anti-tumor immune response and promotes tumor cells killing (37). Accordingly, numerous clinical trials highlighted that TLR-7/9 agonists administration, individually or in combination with ICB therapies, has the potential to stimulate specific T cell response and subsequent tumor regression (38, 39), even for patients with MM (37). More recently, the administration of STING agonists has resulted in tumor regression in mice and generated local and systemic anti-tumor immune responses, as well (40, 41). Clinical trials (phase I) combining STING agonists with ICB are ongoing (Clinicaltrials.gov study identifiers: NCT03843359, NCT04144140 and NCT03010176) for patients with advanced solid tumors or lymphomas. Finally, pDCs-based cancer vaccines have been shown to increase the frequency of circulating anti-tumor T lymphocytes, together with the induction of I-IFN signature in patients with MM (42–44).

In Cutaneous Melanoma (CM), pDCs infiltrate the primary tumor (45) and draining lymph-nodes (46, 47). However, the pDC compartment decreases over disease progression and collapses in metastatic melanoma (MM) patients (34, 37, 46, 48). Moreover, the residual blood pDC component in MM is defective in IFN-α production and displays immunosuppressive features (34, 49). These findings suggest that CM progression is, at least in part, supported by pDCs specific immune escape mechanisms (50). We have recently proposed lactic acidosis as the oncometabolite implicated in pDCs functional impairment in MM patients (49). Here, we investigated the molecular trajectory of pDCs after their exposure to CM cell lines supernatants (SN-mel). IFN-α production by TLR-7/9 and cGAS-STING activation were significantly reduced, suggesting a widespread impairment of PRR pathways in pDCs. By using RNA sequencing, we unveiled that melanoma secretome rewired the pDC transcriptomic profile towards an IFN-defective tolerogenic state, likely dependent of immunosuppressive cytokines and metabolic drift. These findings should better guide windows of intervention for novel immunotherapy approaches empowering the innate compartment of primary cutaneous melanoma (PCM).

## Materials and methods

### Human tissue samples and immunohistochemistry

Tissues were represented by a cohort of 101 PCM, 60 benign nevi (NV) and 5 lupus erythematosus (LE) skin biopsies. Clinical and pathological features of the PCM cases are reported in our previous study (46). The local ethics committee provided formal approval to this project (WV-Immunocancer 2014 to WV, institutional review board code NP906).

Two-to-four micron-thick tissue sections were obtained from formalin-fixed, paraffin-embedded (FFPE) blocks and used for immunohistochemistry. For immunohistochemical staining endogenous peroxidase was blocked by incubation with methanol and hydrogen peroxide 0.03% for 20 minutes during rehydration. Immunostaining was performed using a set of primary antibodies listed in **Supplementary Table S1**. The reaction was revealed using Novolink Polymer (Leica Microsystems) followed by diaminobenzidine (DAB, Dako, Glostrup, Denmark) or using Mach 4 MR-AP (Biocare Medical, Concord, CA, USA), followed by StayRed/AP (Abcam). Finally, the slides were counterstained with Meyer’s Haematoxylin. For double staining, after completing the first immune reaction, the second was visualized using Mach 4 MR-AP (Biocare Medical), followed by Ferangi Blue (Biocare Medical). Quantitative image analysis was performed by using AperioScanscope CS (Aperio, Nikon).

### RNAscope

To localize TGF-β and IL-10 positive cells, tissues were analyzed with RNAscope assay (Advanced Cell Diagnostics, Newark) using RNAscope 2.5 HD Assay-RED kit. The Hs-IL10 probe (cat no. 602051) recognizes the nt 122–1,163 of the IL-10 mRNA (reference sequence NM_000572.2), and the Hs-TGFB1 probe (cat. no. 400881) recognizing the nt 170–1,649 of the TGF-β mRNA (reference sequence NM_000660.4). The sections from fixed human tissue blocks were treated following the manufacturer’s instructions. Briefly, freshly cut 3-mm sections were deparaffinized in xylene (Bio-Optica, cat. no. 06- 1304F) and treated with the peroxidase block solution (ACD, cat. no. 322335) for 10 minutes at room temperature followed by the retrieval solution for 15 minutes at 98°C and by protease plus (ACD, cat. no. 322331) at 40°C for 30 minutes. Hs-POLR2a-C2 (cat. no. 310451) and dapB-C2 (cat. no. 310043-C2) were used as control probes. The hybridization was performed for 2 hours at 40°C. The signal was revealed using RNAscope 2.5 HD Detection Reagent and FAST RED.

### *In situ* PLA

Proximity ligation assay (PLA) was performed using NaveniBright HRP kit (Navinci Diagnostics) according to the manufacturer’s protocol using the following primary antibodies: anti-cGAS (clone D1D3G, #15102, 1:100 pH9, Cell Signaling) and anti-DNA double stranded (clone AE-2, MAB1293, 1:100 pH9, Abcam). Negative controls were performed using only one primary antibody. Slides were analyzed under a Zeiss Axioscope A1 and microphotographs were collected using a Zeiss Axiocam 503 Color with the Zen 2.0 Software (Zeiss). Sections were subsequently immunostained to detect the expression of the E2.2 antigen. IHC was developed using SignalStainBoost IHC Detection rabbit (cod. #18653, Cell Signaling Technology) alkaline phosphatase-conjugated produced in horse and Vulcan Fast Red as substrate chromogen. Again, the sections were analyzed and photographed using Zeiss Axioscope A1 and Zeiss Axiocam 503 Color with the Zen 2.0 Software (Zeiss). Segmented images were obtained using HALO image analysis software (v3.2.1851.229, Indica Labs). HALO image analysis software was used to quantify the PLA signals in twelve non-overlapping fields at high-power magnification (400X) and the output was expressed as “percentage positive cells”.

### Cell cultures

Melanoma cell lines (MCLs), kindly provided by Michele Maio’s laboratory (University Hospital of Siena), were validated as previously reported (46). For the generation of melanoma-conditioned medium (melanoma supernatant, SN-mel), MCLs (designated as Mel146, Mel252, Mel327, and Mel336) were cultured as previously described (46). Mycoplasma contaminations were excluded by routinely testing with Universal Mycoplasma detection kit (Manassas, VA; cat. No. 30-1012K ATCC) according to manufacturer’s suggestions.

### Isolation, culture, and stimulation of human peripheral blood pDCs

Peripheral blood mononuclear cells (PBMCs) were obtained from buffy coats of Healthy Donors (HD) (courtesy of the Centro Trasfusionale, ASST Spedali Civili, Brescia) by Ficoll gradient. Peripheral blood pDCs were magnetically sorted with the Plasmacytoid Dendritic Cell Isolation Kit II (Miltenyi Biotec; cat. No. 130-097-415) and the pDCs purity was greater than 92% for all experiments and greater than 95% for bulk RNA-Sequencing experiments. Blood pDCs (5 x 10^5^ cells/mL) were cultured for 24 hours in RPMI 1640 (Gibco; cat. No. 31870-025) with 10% FBS (Biospa; cat. No. S1810-500), Glutamine and 1% penicillin-streptomycin (Gibco; cat. No. 15070-063) or with the SN-mel, with the addition of human IL-3 (20 ng/mL; Miltenyi Biotec; cat. No. 130-095-071). pDCs were stimulated with R848 (5 μg/mL; Invivogen; cat. No. tlrl-r848-5), CpG-ODN 2216 (6 μg/mL; Miltenyi Biotec; cat. No. 130-100-244) or ADU-S100 (50 μg/mL; Invivogen; cat. No. TLRL-NACDA2R). Brefeldin A (1 μg/mL; Sigma; cat. No. B7651) was added after stimulation to block protein secretion and evaluate intracellular cytokines.

### Flow cytometry analysis

Fluorescence minus one (FMO) was used to set the marker for positive cells. A baseline fluorescence control was used as a reference to set the fluorescence thresholds for positivity. The results were expressed as the percentage of positive cells or as the mean of fluorescence intensity (MFI) of positive cells. To evaluate the IFN-α and CXCL10 intracellular production, pDCs were firstly surface labelled with anti-BDCA-2 (Miltenyi Biotec; cat. No. 130-113-192) and anti-CD123 (Miltenyi Biotec; cat. No. 130-115-270) fluorochrome-conjugated antibodies. Then, pDCs were fixed and permeabilized using the Inside Stain Kit (Miltenyi Biotec; cat. No. 130-090-477) and intracellular staining was performed using the anti-IFN-α (Miltenyi Biotec; cat. No. 130-116-874) and anti-CXCL10 (Biolegend; cat. No. 519504) fluorochrome-conjugated antibodies. Samples were processed on MACS Quant Analyzer 16 (Miltenyi Biotec). Results were analyzed by FlowJo X software (TreeStar Inc., Wilmington, NC, USA).

### ELISA

After 72-hours culture, MCL supernatants were collected and spin at 10000xg for 5 minutes to remove cell debris. IL-10, TGF-β1, and TGF-β2 concentration were determined by using the Human IL-10 DuoSet^®^ ELISA (R&D Systems, cat. No. DY217B), Human TGF-β1 DuoSet^®^ ELISA (R&D Systems; cat. No. DY240), and Human TGF-β2 DuoSet^®^ ELISA (R&D Systems; cat. No. DY302), respectively, following the manufacturer’s instructions.

At 24 hours post-stimulation (as described in the *Isolation, culture, and stimulation of human peripheral blood pDCs* section), pDC supernatants were collected, as described above. The amounts of secreted IFN-α and CXCL10 were determined by using the Human IFN-α Matched Antibody Pair (eBioscience; cat. No. BMS216MST) and Human CXCL10/IP-10 DuoSet^®^ ELISA (R&D Systems; cat. No. DY266). The supernatants were stored at -20°C until use.

### Western blot

pDCs were cultured as described in the *Isolation, culture, and stimulation of human peripheral blood pDCs* section. pDCs were stimulated with ADU-S100 (50 μg/mL; Invivogen; cat. No. TLRL-NACDA2R) for 4 hours. After stimulation, cells were lysed in RIPA lysis buffer (Thermo Fisher Scientific, cat. No. 89900) supplemented with Protease Inhibitor Cocktail (Sigma-Aldrich, cat. No. 78440) and incubated on ice for 20 minutes. Cell lysates were centrifuged at 13000xg for 15 minutes and the suspension was collected and stored at -20°C. Protein concentration was determined by Bradford assay and 20 µg of total proteins were loaded on 4–12% NuPAGE^®^ Bis-Tris Mini Gels (Invitrogen; cat. No. NP0335) under reducing condition and transferred onto a PVDF membrane (Invitrogen, cat. No. LC2007). Membranes were blocked with 5% milk (Biotium, Fremont, CA; cat. No. 22012) in TBS-T (TBS with 0.05% Tween 20; Invitrogen; cat. No. 28360) for 1 hour at room temperature. Primary antibodies were incubated o/n at 4°C in TBS-T with 5% BSA (Sigma-Aldrich, cat. No. A3059). Primary antibodies are listed in **Supplementary Table S1**. The anti-rabbit (Thermo Fischer Scientific; cat. No. 31460) or anti-mouse (Cell Signaling Technologies; cat. No. 7076) secondary antibodies conjugated with horseradish peroxidase were incubated for 1 hour at room temperature. Detection was performed using the SuperSignal™ West Pico Chemiluminescent Substrate (Thermo Fisher Scientific, cat. No. 34577) and visualized by autoradiography.

### Extracellular flux analysis

Glycolytic activity of the pDCs was determined using the Seahorse XFe24 Extracellular Flux Analyzer (Agilent, Santa Clara, CA, USA). Isolated pDCs were cultured in RPMI or SN-mel for 24 hours as described above (*Isolation, culture, and stimulation of human peripheral blood pDCs* section) (Vescovi 2019), washed, and resuspended in Seahorse XF RPMI Medium pH 7.4 (Agilent) by adding 10 mM glucose, 2 mM L-glutamine, 1 mM sodium pyruvate and 20 ng/mL IL-3. Cells were seeded at pre-determined cell density of 200,000 cells/well in 100 µL/well on Seahorse XFe24 V7 PS Cell Culture Microplate (Agilent) pre-coated with poly-D-lysin (Sigma Aldrich, St. Louis, Missouri). Particularly, 50 µL/well of poly-D-lysin 0.1 mg/ml solution were incubated 30 minutes at 37°C, then removed and washed twice. Microplates were centrifuged once at 200× g for 1 min with the break off to aid uniform cell attachment. The plates were transferred to a 37°C incubator not supplemented with CO_2_ for 30 minutes to facilitate cell adhesion. The medium volume was brought up to 500 µL in each well and the plates were return to the incubator for 15 minutes before running the assay. We applied the Seahorse XF Glycolytic Rate Assay (Agilent) to measure real-time extracellular acidification rate (ECAR) and oxygen consumption rate (OCR) of cells and determine the glycolytic proton efflux rate (glycoPER) that is the rate of protons extruded into the extracellular medium during glycolysis (discounting the effect of CO_2_-dependent acidification). The glycoPER is obtained using the following formula: [ECAR (mpH/min) x Buffer Factor (mmol/L/pH) x Geometric Volume (µL) x Volume scaling factor (Kvol)] – CO_2_ Contribution Factor. Following the manufacturer’s instructions, the protocol was set to measure first basal glycolysis in the absence of inhibitors/stimuli. The acute injection effects of the R848 (5 µg/mL) on glycolysis (induced glycolysis) was evaluated after 4 cycles of measurements. Subsequently, the injection of a mixture of the respiratory complex I inhibitor rotenone (Rot; 0.5 µM) and the complex III inhibitor antimycin A (AA; 0.5 µM) was performed to inhibit mitochondrial activity and therefore CO_2_-derived proton production to retrieve compensatory glycolysis. Finally, the glucose analogue 2-deoxy-D-glucose (2-DG; 50 mM) was injected to inhibit glycolysis by blocking glucose hexokinase and exclude other sources of extracellular acidification.

### RNA isolation

2 x 10^5^ pDCs (n = 4) were 24h-cultured with RPMI complete medium or SN-mel (as described in the *Isolation, culture, and stimulation of human peripheral blood pDCs* section), unstimulated or stimulated with R848 (5 μg/mL; Invivogen; cat. No. tlrl-r848-5) for 2 hours. Total RNA was isolated by using AllPrep DNA/RNA/miRNA kit (Qiagen, cat. No. 80224) following manufacturer’s instructions. The optional Dnase I step was included to prevent genomic DNA contamination.

Quantitation and integrity of RNA samples were determined by using the Qubit RNA HS Assay Kit (Life Technologies, cat. No. Q33230) and the RNA 6000 Nano Kit on a Bioanalyzer (Agilent Technologies, Santa Clara, CA, USA; cat. No. 5067-1511), respectively. RNA samples with RIN > 6 were further processed for RNA-sequencing.

### RNA-sequencing analysis

The TruSeq Stranded mRNA Library Prep kit (Illumina, San Diego, CA, USA; cat. No. 20020595) was used to prepare RNAseq libraries from 100ng RNA, after poly(A) capture and according to manufacturer’s instructions. Quality and size of RNA-Seq libraries were assessed by capillary electrophoretic analysis with the Agilent 4200 Tape station (Agilent Technologies) and were quantified by real-time PCR against a standard curve with KAPA Library Quantification Kit (KapaBiosystems). Libraries were pooled at equimolar concentration and sequenced using 75SR reads using Illumina NextSeq 500 platform, generating ~33 million fragments on average per sample.

RNA-seq reads quality was assessed through FastQC software (http://www.bioinformatics.babraham.ac.uk/projects/fastqc/). Reads were clipped from adapter sequences using Scythe software (https://github.com/vsbuffalo/scythe) and low-quality ends (Q score < 20 on a 10-nt window) were trimmed using Sickle (https://github.com/vsbuffalo/sickle). RNA-seq reads were aligned with HISAT2 (https://ccb.jhu.edu/software/hisat2/index.shtml) to the GRCh38 human reference genome. Gene counting was performed using function summarize. Overlaps from GenomicAligments (51) with parameters of “mode = ”Union”, singleEnd = TRUE, ignore.strand = TRUE. Genes expression counts were used for differential gene expression analysis, with the use of DESeq2 package (52). Raw gene expression counts were used for differential gene expression analysis with the DESeq2 package (52). Genes showing an adjusted p-value ≤ 0.05 and |log2FoldChange ≥ 1| were assigned as differentially expressed. Principal component (PC) analysis, by PCA tools package, was applied on the coding transcriptome after applying a ‘regularized log’ transformation of gene expression by DESeq2 package (52). Pre-ranked Gene Set Enrichment Analysis (GSEA) (https://www.gsea-msigdb.org/gsea/downloads.jsp) was performed on pre-ordered ranked gene lists ranked according to the log-normalized fold changes from DESeq2 (53, 54). Gene-sets of Hallmarks, Biocarta, Kyoto Encyclopedia of Genes and Genomes (KEGG), Reactome and Gene Ontology (GO) from MsigDB 7.2 databases (Molecular Signatures Database; https://www.gsea-msigdb.org/gsea/msigdb) were used. GSEA results were filtered by the False Discovery Rate (FDR) < 0.25 and by the enrichment score (ES) greater than 0.5 or lower than -0.5. Heatmap reporting NES values of GSEA analysis have been generated with the seaborn package (python). Upstream regulator analysis (URA) was performed with Ingenuity Pathway Analysis (IPA) software (Qiagen Bioinformatics). Genes showing a |adjusted p-value| < 0.05 and a |Log_2_FoldChange| > 1 were assigned as differentially expressed. Differentially expressed genes (DEGs) that were absent in the IPA database were excluded from the analysis. Results were filtered by overlap p‐value < 0.05 and activation z-score greater than 2 or lower than -2.

### Data processing of the TCGA datasets and statistical analyses

Normalized RSEM counts, by the upper quartile method, for primary solid tumor samples of six TCGA projects (TCGA-BLCA, TCGA-COAD, TCGA-HNSC, TCGA-LUAD, TCGA-LUSC, TCGA-SKCM) (55) were downloaded from Xena portal and merged with metadata of TCGA samples, downloaded using TCGA biolinks R/Bioconductor package (n=2637 cases). For downstream analysis the log2(norm.count +1) gene expression was considered. The estimation of the enrichment score of 6 gene sets of interest (**Supplementary Data 1**) was performed by Gene Set Variation Analysis (GSVA), using the GSVA package (53, 56) and setting the following parameters: min.sz=5, max.sz=2000, kcdf=“Gaussian”, mx.diff=F, parallel.sz=1. In particular, PDC signature was taken from our previous work (46) and I-IFNs/III-IFNs signature was created based on the known genes encoding for interferon-α, -β and –λ. The signatures of genes up-regulated in response to IFN-α protein (designated as *Interferon Alpha Response*) and to TGFβ1 (designated as *TGF-β Signaling*) correspond to the Hallmark gene sets from the Molecular Signatures Database (MSigDB) collections. The signatures of *Interleukin 10 Signaling* and *STING Mediated Induction Of Host Immune Responses* were derived from the Reactome Canonical Pathway database (MSigDB). Moreover, the expression levels of single gene transcripts (IFNA1, IFNL1, IFNG, IL1A, IL1B, IL6, IL10, TNF, TGFB1, TGFB2, PTGES2, CSF1, MxA) were taken into account.

Qualitative variables were described as absolute and relative frequencies; standard descriptive statistics were used for continuous variables, expressing means, standard deviations, medians, and ranges. Comparisons were tested by Mann–Whitney U test or Kruskal-Wallis test, as appropriate. Correlations between quantitative variables were computed using Spearman correlation coefficient. Bonferroni’s correction of significance level was applied for multiple testing in each analysis. Considering the enrichment scores a dimensionality reduction was performed by UMAP, setting the following parameters: n_components = 2, n_neighbors = 15, init = ‘PCA’, learning_rate = ‘0.5’; min_dist = 0.01, metric = ‘euclidean’, random_state = 2 (57). All statistical tests were two-sided, and p-value < 0.05 was considered statistically significant. Unpaired student’s T test and one-way ANOVA were used to compare two and three or more groups, respectively. GraphPad Prism Software version 5 (GraphPad Software, San Diego, CA, USA), R (version 4.0.2) and R studio) were used for statistical analysis and rendering graphs.

## Results

### pDC infiltration and activation is impaired in advanced PCM

**Figure 1:**
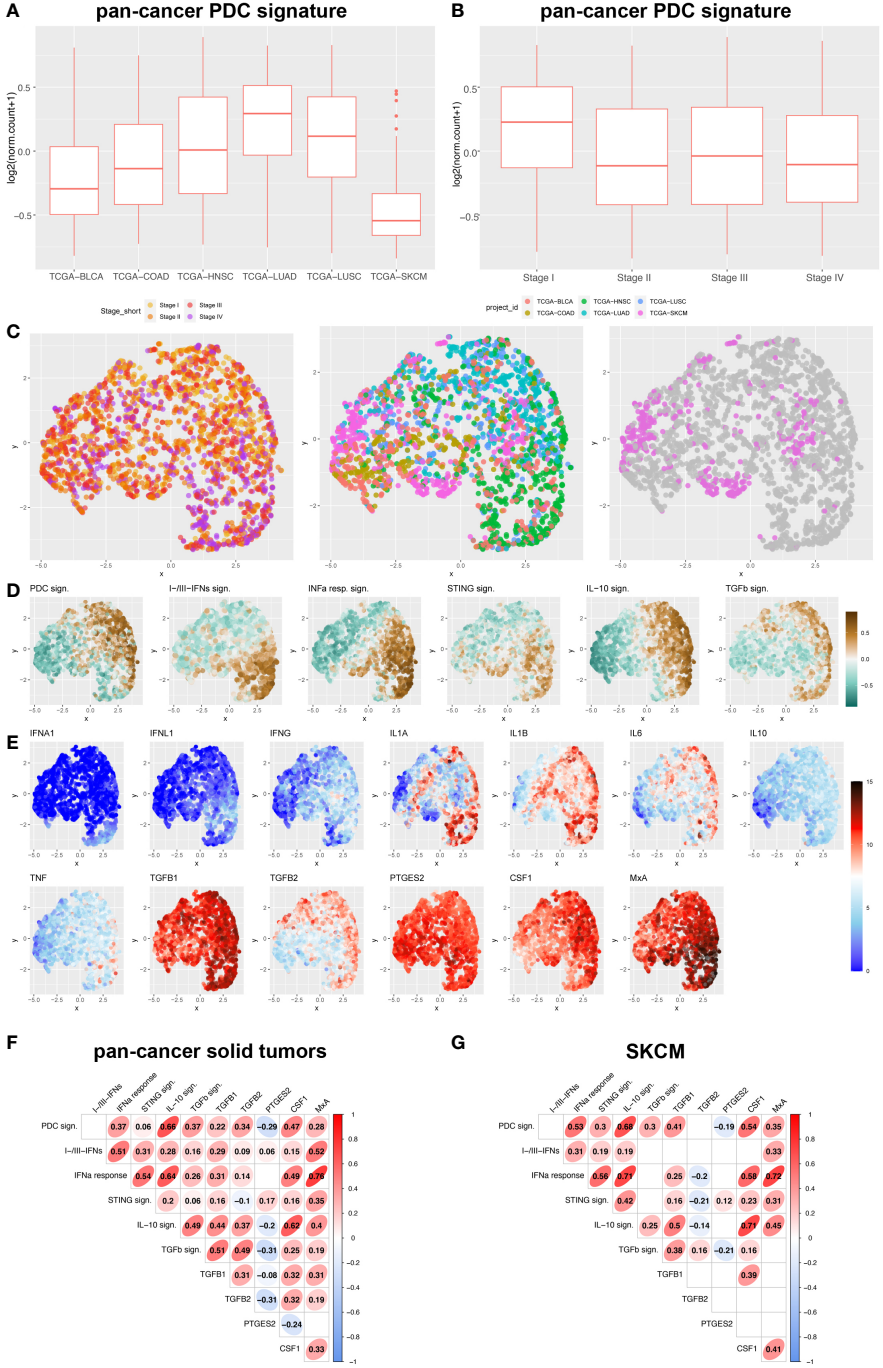
pDC and interferon-related signatures and cytokines expression across human pan-cancers solid tumors and melanoma. **(A, B)** Box plots representing PDC signature among different solid tumor sites **(A)** and stages **(B)** (BLCA, bladder cancer; COAD, colorectal cancer; HNSC, Head&Neck squamous cell carcinomas; LUAD, lung adenocarcinomas; LUSC, lung squamous cell carcinomas; SKCM, skin cutaneous melanomas). **(C-E)** UMAP plots showed expression distribution of pDC signature, I-IFNs/III-IFNs signature, IFN-α response signature (corresponding to *Hallmark_Interferon Alpha Response* in MSigDB), STING signature (corresponding to *Reactome_STING Mediated Induction Of Host Immune Responses* in MSigDB), IL-10 signaling signature (corresponding to *Reactome_Interleukin 10 Signaling* in MSigDB), TGF-β signaling signature (corresponding to *Hallmark_TGF-β Signaling* in MSigDB) **(D)**, and expression of 12 gene transcripts encoding for pro-inflammatory and immunosuppressive cytokines (IFNA1, IFNL1, IFNG, IL1A, IL1B, IL6, IL10, TNF, TGFB1, TGFB2, PTGES2 and CSF1) **(E)** among different stages of primary solid tumors (I to IV) and different anatomic origins (BLCA, bladder cancer; COAD, colorectal cancer; HNSC, Head&Neck squamous cell carcinomas; LUAD, lung adenocarcinomas; LUSC, lung squamous cell carcinomas; SKCM, skin cutaneous melanomas) **(C)**. **(F, G)** Correlograms showing the Spearman correlation between each signature in pan-cancer primary solid tumors **(F)** and SKCM **(G)** by exploring TCGA datasets. R coefficient is shown and ellipses indicate significant results (p < 0.05).

pDCs infiltrate a wide set of human cancers (37), including melanoma. We extended this finding by analyzing a pan-cancer TCGA datasets (**Supplementary Table S2**) and measuring the pDC content using a previously identified signature (46). pDC signature resulted poorly expressed in skin cutaneous melanoma (TCGA-SKCM) as compared to primary carcinomas (TCGA-BLCA, TCGA-COAD, TCGA-HNSC, TCGA-LUAD, TCGA-LUSC; **Figure 1A**; **Supplementary Table S3**). Moreover, a high pDC content was limited to draining lymph nodes (46) and stage I primary tumors (**Figure 1B**; **Supplementary Table S4**).

**Figure 2:**
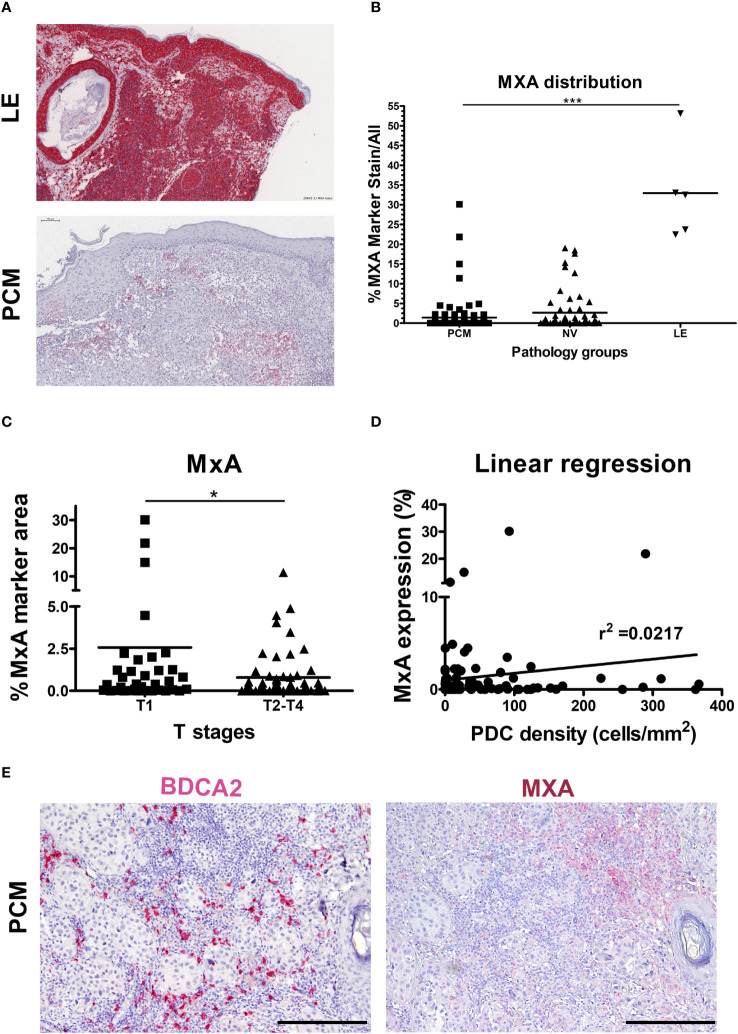
Limited MxA expression in human PCM. **(A)** Representative sections of lupus erythematosus (LE; upper panel) and primary cutaneous melanoma (PCM; lower panel) biopsies showing high and low levels of MxA expression, respectively. Sections are stained as label and counterstained with Haematoxylin. Magnification 100X. Scale bar 100 µm. **(B)** Dot plots show the percentages of MxA positive signal on evaluated area in PCM, benign nevi (NV) and LE biopsies. Bars represent the mean of biological replicates. The statistical significance was calculated by One-way ANOVA (p< 0.0001) and Bonferroni multiple comparison; ***p < 0.001. **(C)** Dot plots show the percentage of MxA^+^ marker area, obtained by digital microscopy analysis, in T1 versus T2-T4 stages of PCM. Bars represent the mean of biological replicates. The statistical significance was calculated by unpaired Student’s T test (p= 0.0411); *p < 0.05. **(D)** Linear regression analysis between MxA expression and BDCA2^+^ pDCs density in PCM. **(E)** Representative images of BDCA2 (left panel) and MxA (right panel) staining in a PCM case. Magnification 100X. Scale bar 200 µm.

We devised signatures interrogating interferons expression (referred as I-IFNs/III-IFNs signature) and response to interferons (referred as IFN-α Response signature) and STING mediated immune response activation (referred as STING signature) as well as expression of other major cytokines (**Figures 1C-E**; **Supplementary Data 1**). We found that IFNA1 and IFNL1 gene transcripts resulted negligibly expressed across the pan-cancer dataset (**Figure 1E**) when compared to other immunosuppressive and pro-inflammatory cytokines such as TGFB1/2, PTGES2, IL1A, IL1B, IL6 and CSF1. However, the PDC signature and IFN-α Response signature directly correlated, especially in the TCGA-SKCM dataset (**Figures 1F, G**). Moreover, IFN-α Response signature directly correlated with I-IFNs/III-IFNs, MxA (human myxovirus resistant protein 1) and STING signatures (**Figures 1F, G**); however, no correlation was detected between pDC and I-IFNs/III-IFNs signatures (**Figures 1F, G**). These findings suggest that pDCs might be partly dysfunctional in type I and III IFNs production and that activation of IFN-α/STING pathway derive from different cellular sources in the CM TME. To further explore the functional impairment of PCM-associated pDCs, we assessed the expression of MxA, as surrogate of I-IFN production, on melanoma biopsies (n=99) (**Figures 2A, B**). Positive controls (58), represented by lupus erythematosus (LE) skin biopsies (n=5), showed a strong and diffuse MxA reactivity in all compartments, including epidermis and dermal structures (**Figures 2A, B**). Although heterogeneous within the PCM cohort, the MxA reactivity was significantly reduced in PCM compared to LE (1.42% vs 32.96%; p < 0.0001), but comparable between PCM and benign nevi (NV) (**Figure 2B**), the latter largely devoid of pDCs (46). MxA was significantly reduced along the PCM progression from T1 to T2-T4 stages (p = 0.04; **Figure 2C**). Moreover, linear regression analysis proved that the lack of MxA expression is not related to a poor pDC density (r^2 = ^0.02; **Figures 2D, E**). All together, these findings propose that defective I-IFN endogenous production by pDCs occur over PCM progression.

### cGAS-STING activation is impaired in PCM-associated pDCs

**Figure 3:**
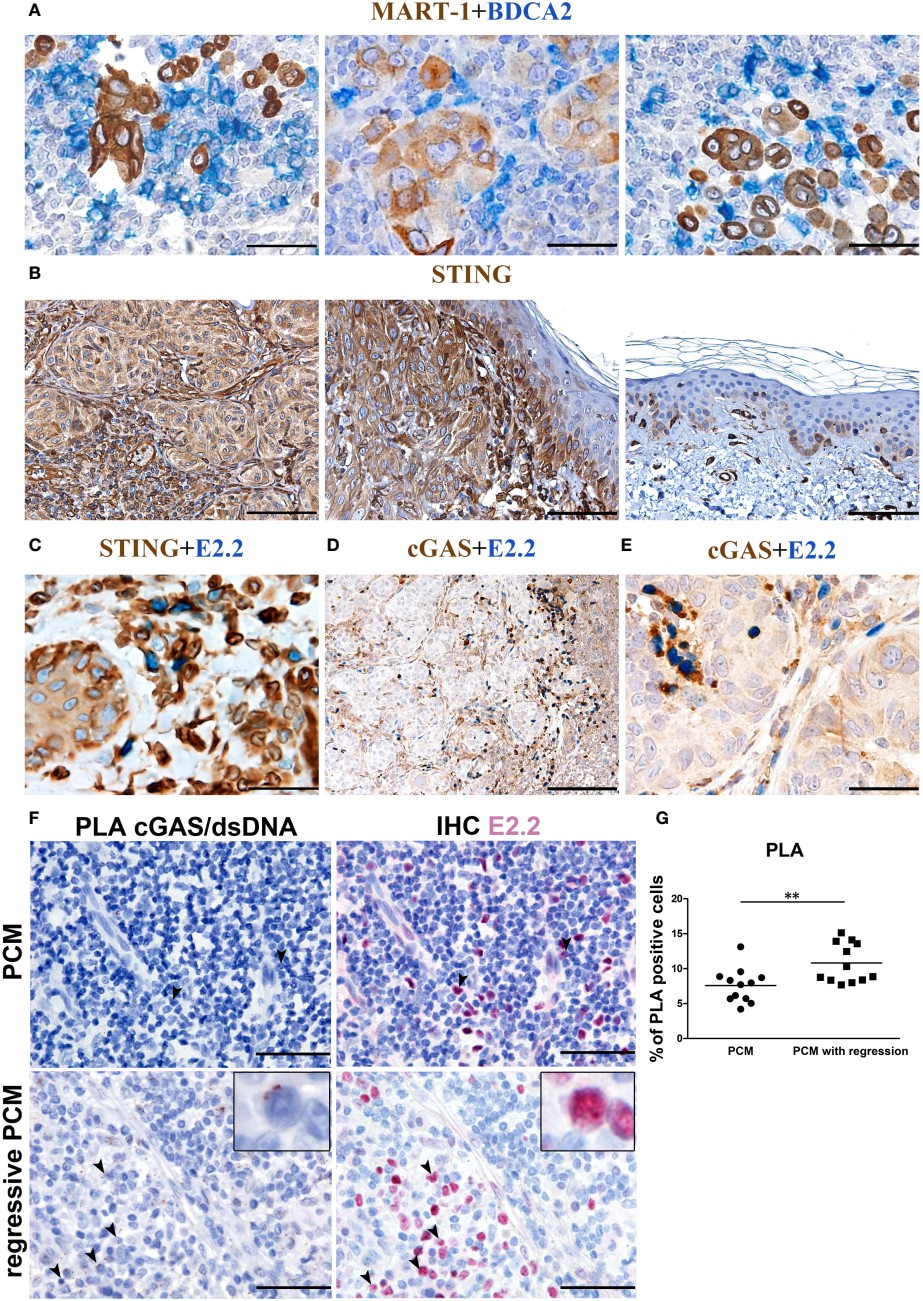
cGAS expression and endogenous activation in PCM-infiltrating pDCs. Representative PCM cases showing **(A)** the interaction between BDCA2^+^ pDCs and Mart-1^+^ melanoma cells, **(B)** STING reactivity that results positive on PCM case and negative on adjacent normal skin **(C)** STING reactivity on E2.2^+^ pDCs and **(D, E)** cGAS reactivity on E2.2^+^ pDCs. Magnification 400X. Scale bar 50 µm **(A, C, E)**. Magnification 100X, scale bar 200µm **(B, D)**. **(F)** Representative microphotographs showing dsDNA-cGAS interactions detected by PLA (brown; left panels) combined with anti-E2.2 IHC staining (red; right panels) in PCM cases (upper panels) and PCM cases with regression (lower panels). Increased signals elements that co-express the dsDNA-cGAS interactions and E2.2 signals is observed in PCM cases with regression, identified as activated pDCs pointed by black arrowheads (zoomed in on the insert). Scale bar 50µm. **(G)** Scatter dot plots show the percentage of PLA positive cells evaluated in twelve non-overlapping fields at high-power magnification (400X) of PCM and PCM with regression cases. Bars represent the mean of biological replicates. The statistical significance was calculated by unpaired Student’s T test (p= 0.0068); ** p < 0.01.

In PCM tissues, pDCs can be found near melanoma cells (**Figure 3A**). Besides TLRs, human pDCs express endosomal and cytosolic nucleic acid sensors and might sense nucleic acids released from dying tumor cells (59–61). Tumor-derived DNA leads to the activation of the cGAS–STING pathway (59). Specifically, cGAS interacts with double-stranded DNA (dsDNA) and induces conformational changes into 2’,3’-cyclic GMP-AMP (cGAMP). The second messenger cGAMP then activates STING (62). The activation of dendritic cells *via* STING pathway is crucial to eradicating tumors in mouse models (40), resulting in a strong priming of antigen-specific T cells with clinical responses (43).

We thus explored the endogenous activation of cGAS-STING pathway (13) in human PCM samples. STING expression resulted diffuse and strong in melanoma cells, overlying keratinocytes, blood vessels and cells of the TME including pDCs (**Figures 3B, C**); on the contrary, no STING reactivity was observed in keratinocytes distant from melanoma nests (**Figure 3B**). cGAS expression resulted more limited, with positive cells represented by a variable fraction of E2.2^+^ pDCs (**Figures 3D, E**), a fraction of melanoma cells (**Figure 3D**) and, as expected (63), infiltrating plasma cells (**Supplementary Figure S1**).

To test endogenous dsDNA-cGAS activation by pDCs infiltrating PCM, we analyzed pDC-enriched PCM cases (n=5) by using proximity ligation assay (PLA) with antibodies anti-cGAS and anti-DNA double stranded. Image analysis in these cases resulted in a very limited signal, and staining with E2.2 confirmed a negligible endogenous cGAS activation in pDCs (**Figure 3F** upper panels and **Supplementary Figure S2A**). However, by exploring PCM cases (n=3) with consistent areas of spontaneous microscopic melanoma regression (64, 65) and a large number of infiltrated E2.2^+^ pDCs, we could detect an increased signal suggesting endogenous pDCs activation (**Figure 3F** lower panels and **Supplementary Figure S2B**). By quantification of PLA signals, we observed a significant increase of dsDNA-cGAS activation in regressive PCM (regressive PCM mean=10.800355 vs PCM mean=7.58688 PLA positive cells/field; **Figure 3G**). These findings suggest a poor cGAS-STING endogenous activation in PCM microenvironment.

### Melanoma secretome impairs the production of IFN-α and CXCL10 by pDCs

**Figure 4:**
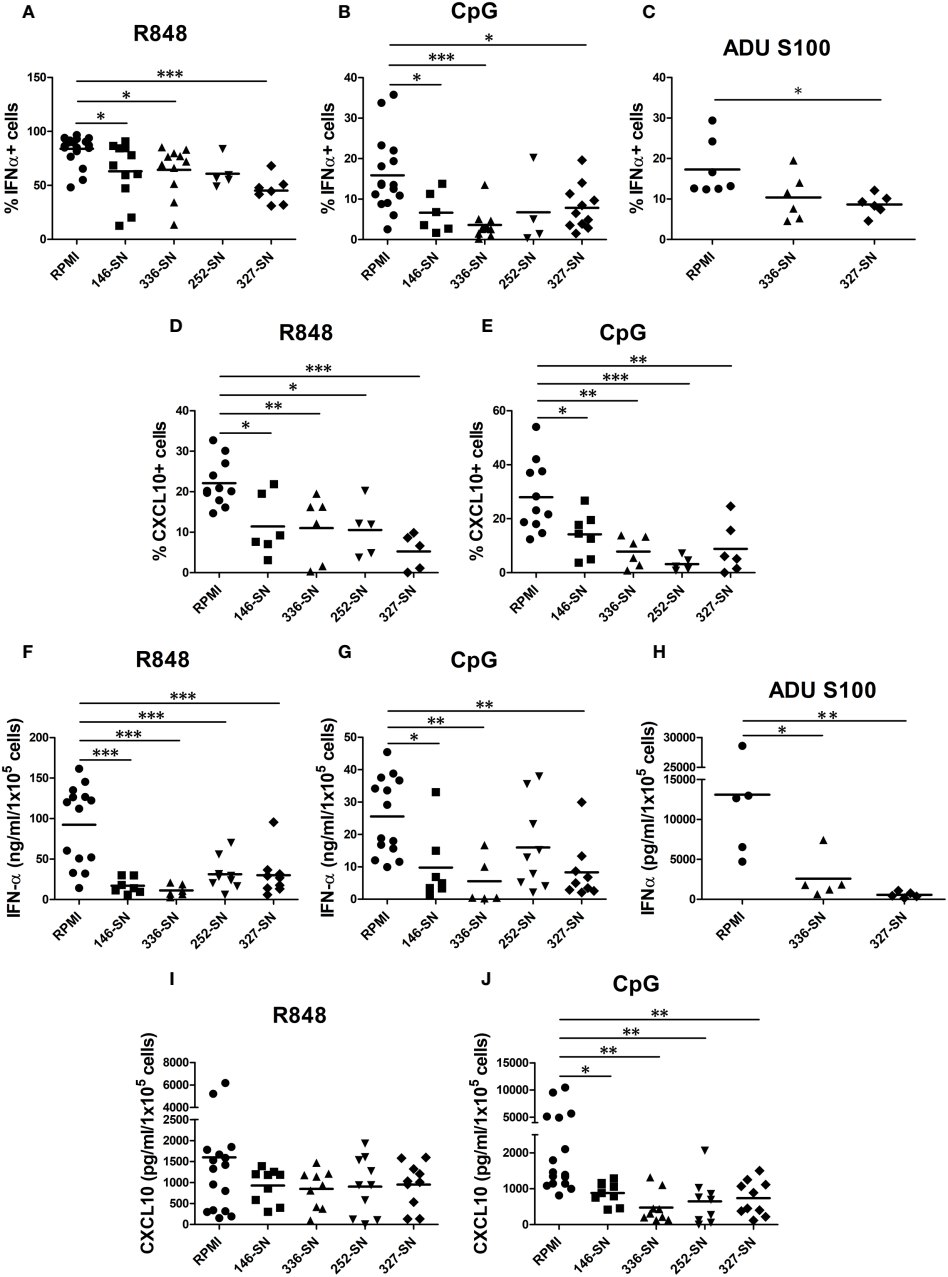
SN-mel impair the IFN-α and CXCL10/IP-10 production by pDCs. pDCs isolated from HD were exposed to SN-mel for 24 h. pDCs were stimulated with R848 for 2 h **(A)**, 6 h **(D)** and 24 h **(F, I)** or with CpG-ODN 2216 or ADU S100 for 6 h **(B, C, E)** or 24 h **(G, H, J)**. Brefeldin A was added 1 h and 4 h prior to 2 h and 6 h stimuli respectively. Both IFN-α and CXCL10 intracellular production **(A-E)** and secretion **(F-J)** were measured by flow cytometry and ELISA, respectively. Scatter dot plots show the percentage of IFN-α or CXCL10 positive cells evaluated on BDCA-2+/CD123+ pDCs **(A-E)** or the protein concentration per 1x10^5^ cells measured on pDC supernatants **(F-J)**. Bars represent the mean of biological replicates. The statistical significance was calculated by One-way ANOVA (A p= 0.048; B p= 0.0007; C p= 0.0264; D p= 0.0003; E p= 0.0001; F p< 0.0001; G p= 0.002; H p=0.009; I p= 0.3; J p= 0.0012) and Bonferroni multiple comparison; * p < 0.05; ** p < 0.01; *** p < 0.001.

In CM tissues, pDCs are exposed to secreted products of melanoma cells and cells of the microenvironment. By intracellular flow cytometry, we tested the proficiency of human circulating pDCs from healthy donors to produce IFN-α and CXCL10 after *in vitro* exposure to supernatant obtained from various MCL (SN-mel). The frequency of IFN-α^+^ pDCs following R848 or CpG stimulation was reduced in every SN-mel and significantly reduced in three out of four (SN-mel146; SN-mel336; SN-mel327) compared to RPMI (**Figures 4A, B**). In parallel, we tested the *in vitro* activation of circulating pDCs by STING agonist. The frequency of IFN-α^+^ pDCs after stimulation with ADU-S100 was significantly diminished by SN-mel (**Figure 4C**). It should be noted that IFNα protein resulted nearly absent (<1.5% IFN-α^+^ pDCs; <2ng/ml IFN-α) at baseline condition; however, their exposure to SN-mel further impairs IFN-α production (**Supplementary Figures S3A-C**). Similarly, the production of CXCL10, a pro-inflammatory cytokines relevant for the recruitment of antigen-specific T-cells into the tumor tissues (66) and mainly dependent on IFNα-IFNAR autocrine/paracrine signaling in pDCs (67), resulted consistently reduced by SN-mel as shown by intracellular flow cytometry (**Figures 4D, E**). Also, MFI of both IFN-α^+^ and CXCL10^+^ pDCs, confirmed a trend towards decreased amounts of expression by SN-mel conditioned cells (**Supplementary Figures S3D-G**). Overall, these data highlight a defective intracellular IFN-α and CXCL10 production in pDCs exposed to melanoma soluble factors. We subsequently confirmed this defect by ELISA. Specifically, upon pDC activation, the IFN-α concentration measured by ELISA was significantly diminished after exposure to supernatants of melanoma cell lines (**Figures 4F-H**). Similarly, the amount of CXCL10 secreted by SN-mel-exposed pDCs was significantly diminished after CpG stimulation; at variance, only a slight decrease of CXCL10 concentration was measured in R848 stimulated pDCs exposed to SN-mel (**Figures 4I, J**). Overall, these results support a role of CM-derived soluble factors in dampening the pDC proficiency to produce both IFN-α and CXCL10 upon activation via TLR7/9 and STING signaling.

### Melanoma secretome imprints immunosuppressive pathways and metabolic drift in pDCs

**Figure 5:**
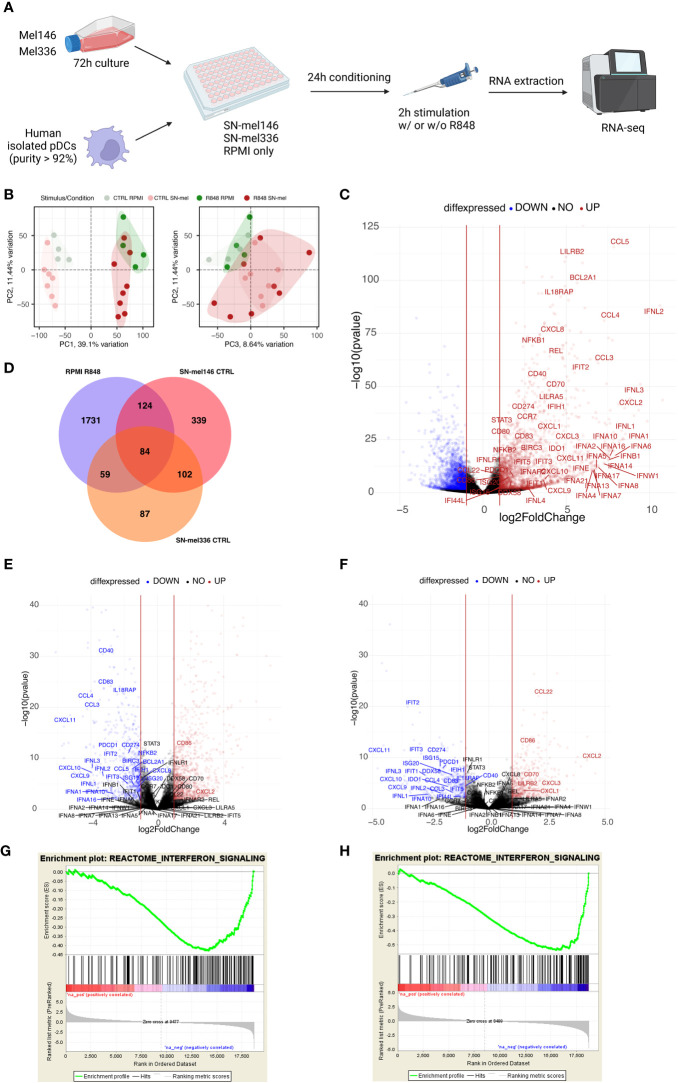
SN-mel modulates the pDC transcriptomic profile toward an immune suppressive state. **(A)** Graphical abstract depicts the experimental settings. Created with BioRender.com. **(B)** Scatter plots showing the combined projections of the first three components of a principal component analysis (PCA) run considering the whole gene expression on the SN-mel-exposed or RPMI-cultured pDCs samples resting and R848 stimulated. Samples’ features are highlighted by colour code. **(C)** Volcano plots showing the log2 Fold Change and -log10 p-value of differential expression among RPMI-R848 stimulated vs RPMI-CTRL unstimulated pDCs. **(D)** Venn diagram shows the number of upregulated genes in R848 stimulated pDCs *versus* unstimulated pDCs (purple) and the number of downregulated genes in unstimulated pDCs exposed to SN-mel 146 (red) and SN-mel 336 (orange) as compared to unstimulated pDCs in RPMI condition. **(E, F)** Volcano plots showing the log2 Fold Change and -log10 p-value of differential expression among SN-mel146-CTRL vs RPMI-CTRL **(E)** and SN-mel336-CTRL vs RPMI-CTRL **(F)** pDCs. Vertical red lines highlight a |Log2FoldChange| = 1. **(G, H)** Enrichment plots for the set of the interferon signaling in the transcriptome of SN-mel146 **(G)** and SN-mel336 **(H)** exposed pDCs versus RPMI cultured pDCs by GSEA.

To further dissect the molecular mechanisms driving pDC functional impairment in CM, we performed bulk RNA sequencing (RNA-seq) analysis of healthy pDCs, cultured in RPMI or in melanoma conditioned medium (SN-mel146; SN-mel336) and either left untreated or stimulated with R848 for 2 hours (**Figure 5A**, **Supplementary Figure S4**). By principal component analysis (PCA), the SN-mel exposed pDCs segregated along the first PCA axis (PC1) into TLR7 activated and non-activated sample groups (**Figure 5B**, **Supplementary Figure S5**). By combining PC2 and PC3 we could identify the subgroups of SN-mel exposed pDCs (burgundy dots) *versus* RPMI cultured pDCs (green dots), being the latter characterized by high PC2 and low PC3 values (**Figure 5B**, **Supplementary Figure S5**). Specifically, SN-mel146 subgroup (red dots) is characterized by higher PC3 and lower PC2 values in comparison to SN-mel 336 (orange dots) (**Supplementary Figure S5C**). By exploring the loadings of each PCs, type I and type III IFNs transcripts resulted strongly and directly related to PC1 and PC2 and inversely related to PC3 (**Supplementary Figure S6**). Moreover, genes encoding for cytokines including IL12A, IL6, and IL27 and effector/memory T cell-attracting chemokines (i.e. CCL3, CCL4, and CCL5) are among the top-fifty PC1 loadings. A set of molecules that are known to modulate the interactions between tumor cells and microenvironment towards a pro-tumoral and immunosuppressive phenotype, such as human lectin CLEC17A (also known as Prolectin) (68, 69), the thrombin receptor FR2 (also known as protease-activated receptor-1, PAR-1) (70–73), the spermine oxidase (SMOX) (74), the transcription factor TOX (75–77) and type II transmembrane glycoprotein ENPP1, were among component loadings inversely related to PC2 as well as directly related to PC3, representing SN-mel conditioning (**Supplementary Table S5**).

As expected, GSEA-based functional enrichment showed that up-regulated genes in R848-stimulated pDCs were enriched for anti-viral response and cytokine response. Particularly, type I IFN-related or -induced pathways were enriched. Thoroughly, TLR7 stimulation induced up-regulation of different subtypes of IFNs and IFN inducible genes, as well as cytokines encoding genes (**Figure 5C**; **Supplementary Table S6**). Additionally, R848 induced expression of co-stimulatory molecules and chemokine receptor CCR7, which is involved in pDC recruitment to the tumor site (78) (**Figure 5C**; **Supplementary Table S6**). Finally, we identified IL18RA, LILRA5, and LILRB2, as surface markers associate to I-IFN production (79) (**Figure 5C**; **Supplementary Table S6**).

We found that a relevant number of transcripts were significantly modulated by SN-mel146 and SN-mel336 as compared with RPMI (**Supplementary Figure S7**). We focused on the lists of differentially expressed genes (DEGs) that were up-regulated in R848 activated pDCs while being down-regulated by SN-mel exposure (**Figure 5D**), as emphasized by volcano plots (**Figures 5C, E, F**). For instance, IFNA as well as IFNL genes expression was significantly down-regulated in SN-mel-exposed pDCs (**Table 1**; **Figures 5E, F**). Additionally, chemokines such as CXCL9, CXCL10 and CXCL11, were significantly down-regulated in SN-mel exposed pDCs (**Table 1**; **Figures 5E, F**). Remarkably, also mRNA expressions of CD274/PD-L1 and PDCD1/PD-1 were significantly down-regulated in pDCs exposed to melanoma secretome (**Table 1**; **Figures 5E, F**) suggesting that pDCs become hyporesponsive to TLR-mediated stimulation when exposed to CM secretome (80). On the other hand, CD86 and CXCL2 were up-regulated by SN-mel (**Figures 5E, F**). To assign biological relevance to DEGs, we run a comprehensive GSEA-based functional analysis. SN-mel-exposed pDCs revealed negative enrichment of functional gene sets related to innate immune response and I-IFN signaling (**Table 2**, **Supplementary Data 2**, **Supplementary Figure S8A**, **Figures 5G, H**). Among metabolic pathways, glycolysis was identified as negatively enriched only in pDCs exposed to SN-mel146 (**Supplementary Figure S8B**) hinting glucose deprivation (81). We further identified positive enrichment of TGF-β signaling in pDCs exposed to SN-mel146 suggesting that melanoma cells might secrete TGF-β (82) (**Supplementary Figure S8C**).

**Table 1:** IFNA and chemokines genes expression in pDCs.

HGNC symbol	R848 vs CTRL		SN-mel 146 vs RPMI		SN-mel 336 vs RPMI		(SN-mel 146 vs RPMI) + R848		(SN-mel 336 vs RPMI) + R848	
	Log_2_FC	p adj	Log_2_FC	p adj	Log_2_FC	p adj	Log_2_FC	p adj	Log_2_FC	p adj
IFNA1	8,46	3,22E-24	-3,00	0,0002	-2,11	0,0124	-1,78	0,0263	-1,89	0,0186
IFNA2	7,64	2,43E-15	-1,12	ns	-0,07	ns	-0,98	ns	-0,83	ns
IFNA4	6,57	4,45E-10	-0,56	ns	0,25	ns	-1,66	ns	-1,12	ns
IFNA5	6,82	3,69E-11	-0,68	ns	0,19	ns	-1,19	ns	-1,17	ns
IFNA6	7,94	1,02E-16	-1,58	ns	-0,61	ns	-2,28	0,0124	-1,74	ns
IFNA7	6,68	1,24E-10	-0,75	ns	0,12	ns	-1,58	ns	-1,05	ns
IFNA8	6,92	1,38E-11	-0,77	ns	0,13	ns	-1,39	ns	-1,04	ns
IFNA10	8,39	1,29E-24	-2,65	0,0008	-2,50	0,0026	-1,40	Ns	-1,10	ns
IFNA13	6,60	2,46E-10	-0,74	ns	0,11	ns	-1,48	ns	-1,19	ns
IFNA14	7,30	2,30E-13	-0,82	ns	0,12	ns	-1,07	ns	-1,03	ns
IFNA16	7,64	1,30E-16	- 2,44	0,0056	-1,39	ns	-1,94	0,0276	-1,44	ns
IFNA17	6,77	1,20E-10	-0,42	ns	0,39	ns	-1,61	ns	-1,17	ns
IFNA21	5,98	5,43E-08	-0,35	ns	0,35	ns	-1,93	ns	-1,20	ns
IFNL1	8,56	1,28E-26	-3,52	7.85E-05	-2,59	1,74E-02	-1,93	ns	-1,52	ns
IFNL2	10,63	6,86E-86	-3,26	5.13E-06	-2,06	1,28E-02	-1,41	3,04E-02	-0,84	ns
IFNL3	9,44	1,63E-49	-3,93	1.87E-07	-3,63	2,41E-05	-1,39	ns	-0,78	ns
IFNL4	2,74	0,037	0,79	ns	0	ns	0.37	ns	-0,54	ns
CXCL9	3,625	7,11E-06	-3,86	3.27E-06	-3,61	1,51E-04	-0,70	ns	-0,83	ns
CXCL10	3,692	3,66E-06	-3,94	1,24E-06	-3,64	1,05E-04	-0,62	ns	-0,35	ns
CXCL11	4,392	3,97E-13	-5,25	3,45E-17	-4,53	1.57E-10	-0,20	ns	-1,05	ns
CD274	3,43	1,13E-39	-1,74	3,84E-10	-2,04	1,42E-10	-0,07	ns	-0,88	0,006
PDCD1	1,81	1,39E-07	-2,27	7,94E-11	-1,99	1,38E-06	-0,21	ns	-0,51	ns
IRF7	0.13	ns	-8.5	1,51E-15	-1,09	7,00E-07	-0,41	ns	-0,47	0.04

Green colour indicates down-regulated genes. Red colour indicates up-regulated genes.

**Table 2:** Interferon-related pathways negatively enriched in SN-mel-exposed pDCs resulting from the Gene Set Enrichment Analysis (GSEA).

	Unstimulated						Stimulated with R848					
	SN-mel 146-exposed pDCs			SN-mel 336-exposed pDCs			SN-mel 146-exposed pDCs			SN-mel 336-exposed pDCs		
ENRICHED PATHWAY	ES	NES	FDR q-val	ES	NES	FDR q-val	ES	NES	FDR q-val	ES	NES	FDR q-val
KEGG_CYTOSOLIC_DNA_SENSING_PATHWAY	-0,58	-2,13	0	-0,58	-1,53	0,24	-0,48	-1,83	0,01	-0,61	-2,49	0
REACTOME_INTERFERON_ALPHA_BETA_SIGNALING	-0,66	-2,37	0	-0,76	-2,56	0	-0,60	-2,38	0	-0,75	-3,21	0
REACTOME_TRAF6_MEDIATED_IRF7_ACTIVATION	-0,65	-1,99	0	-0,58	-1,71	0,03	-0,66	-2,22	0	-0,73	-2,64	0
REACTOME_REGULATION_OF_IFNA_SIGNALING	-0,65	-1,97	0	-0,60	-1,68	0,04	-0,76	-2,49	0	-0,78	-2,61	0
REACTOME_INTERFERON_SIGNALING	-0.43	-1,80	0,01	-0,54	-2,03	0	-0,32	-1,49	0,11	-0,46	-2,20	0
REACTOME_RIG_I_MDA5_MEDIATED_INDUCTION_OF_IFN_ALPHA_BETA_PATHWAYS	-0,44	-1,65	0,05	-0,47	-1,60	0,07	-0,41	-1,66	0,04	-0,55	-2,42	0
GO_RESPONSE_TO_TYPE_I_INTERFERON	-0,63	-2,36	0	-0,73	-2,41	0	-0,56	-2,16	0	-0,73	-3,15	0
GO_REGULATION_OF_TYPE_I_INTERFERON_MEDIATED_SIGNALING_PATHWAY	-0,59	-1,99	0,03	-0,55	-1,73	0,18	-0,64	-2,30	0	-0,66	-2,52	0

To better understand the molecular mechanisms responsible for the transcriptional rewiring of SN-mel-exposed pDCs, we performed upstream regulator analysis (**Table 3**). Interestingly, the positive upstream regulators included TAB1 that is a signaling mediator between TGF-β receptor and the kinase TAK1. On the other hand, we recognized many negative upstream regulators implicated in response to damaged- or pathogen-associated molecular patterns (DAMPs and PAMPs) and IFNs signaling (e.g. TLR7 and IRF7; **Table 3**). Overall, bulk transcriptomic findings indicate that soluble components produced by melanoma cells, including immunosuppressive cytokines and metabolites, significantly impair pDC activation.

**Table 3:** Upstream regulators in SN-mel-exposed pDCs predicted to be inhibited or activated based on IPA analysis.

UPSTREAM REGULATORS	SN-mel 146-exposed pDCs		SN-mel 336-exposed pDCs	
	Activation z-score	p-value of overlap	Activation z-score	p-value of overlap
BTK	3,031	6,41E-11	4,201	1,33E-24
IL1RN	3,326	8,37E-06	5,009	1,64E-21
TAB1	3,317	6,9E-0,3	2,137	2,67E-10
MAPK1	3,263	1,84E-06	5,939	2,26E-21
MAPK2K3	-2	2,25E-03	-2,236	6,07E-06
MAPK2K6	-2	1,20E-03	-2,229	2,36E-06
IFNB1	-2,113	1,44E-03	-3,349	2,38E-12
IFNAR2	-2,236	6,9E-0,3	-2,236	3,59E-0,4
NONO	-2,236	6,92E-03	-2	3,56E-03
IL27	-2,335	8,01E-10	-2,132	5,70E-10
TLR7	-2,388	1,92E-10	-2,687	2,55E-25
TICAM1	-2,449	1,63E-04	-2,236	7,47E-05
PRL	-2,457	1,75E-05	-5,992	1,73E-39
IRF3	-2,694	6,32E-06	-2,54	3,27E-08
TBK1	-2,774	1,55E-06	-2,219	1,17E-04
PDLIM2	-2,837	5,84E-06	-2,84	3,47E-06
IRF7	-2,951	4,80E-06	-2,798	1,66E-07
STAT1	-3,285	1,20E-11	-4,277	2,44E-25
Interferon alpha	-3,385	2,11E-12	-5,094	2,50E-44
IRF1	-3,575	1,07E-11	-2,621	8,24E-08
PAF1	-3,606	7,57E-05	-4,243	2,42E-13
NFkB (complex)	-3,645	1,17E-14	-2,043	4,44E-09
IFNA2	-4,038	8,37E-06	-5,868	5,22E-33
IFNL1	-4,124	3,47E-09	-6,27	3,68E-41
TLR9	-4,14	1,01E-10	-4,798	2,71E-24
IFNG	-4,2	4,9E-18	-4,856	3,69E-36
TNF	-4,342	1,08E-23	-2,167	7,50E-16

### Functional impairment of pDCs is mediated by immunosuppressive cytokines *via* IRF7 downregulation, while IRF3 activation is preserved

**Figure 6:**
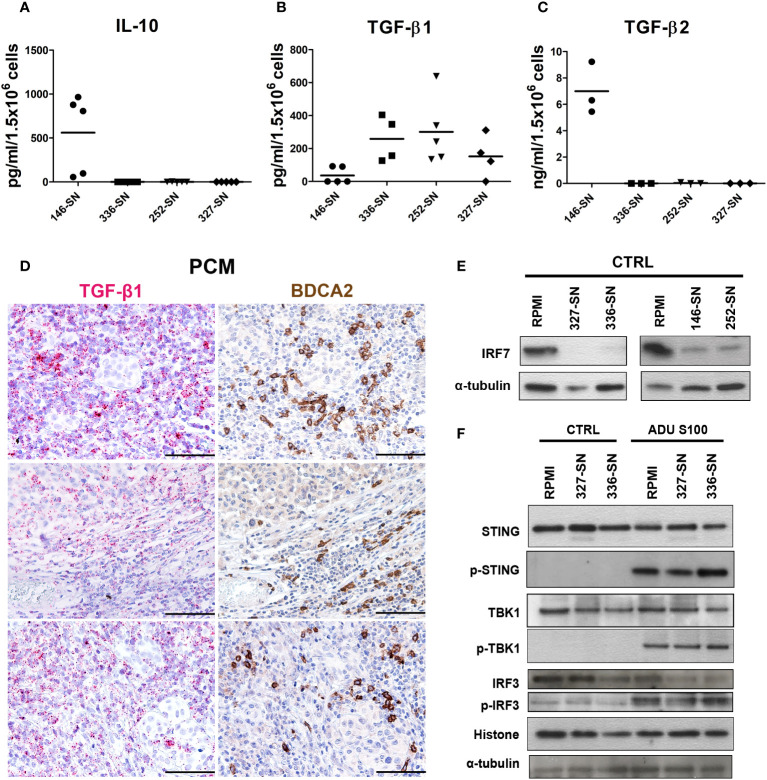
IL-10 and TGF-β production by melanoma cells and concomitant IRF7 downregulation, but preserved STING signaling, in melanoma-conditioned pDCs. IL-10 **(A)**, TGF-β2 **(B)**, and TGF-β1 **(C)** secretion by melanoma cell lines was measured by ELISA on SN-mel collected after 72 h of culture. Scatter dot plots show the concentration of cytokines per 1.5x10^6^ seeded cells. Bars represent the mean value of biological replicates (n =3-5). The statistical significance was calculated by one-way ANOVA (A p= 0.0019; B p< 0.0001; C p= 0.0508) and Bonferroni’s multiple comparison test. **(D)** Representative images of TGF-β RNAscope (left panels) and BDCA2 staining (right panels) in PCM cases. Magnification 200X. Scale bar 100 µm. **(E)** Immunoblots showing IRF7 expression in pDCs cultured in RPMI medium or SN-mel for 24 h, as labeled. α-tubulin was used as housekeeping control. **(F)** Immunoblots showing STING, TBK1, and IRF3 expression and their phosphorylation p-STING (Ser366), p-TBK1 (Ser172), and p-IRF3 (Ser396) in pDCs cultured in RPMI or SN-mel as labeled. Histone H3 and α-tubulin were used as housekeeping control.

Tumor cells, including melanoma cells, produce and release a broad set of immunosuppressive cytokines (83–87). A diminished capacity of pDCs to produce IFN-α upon TLR7/9 stimulation has been previously reported in HNSCC (29), breast (88) and ovarian cancers (89). Transforming growth factor β (TGF-β) is abundant in these cancers and has been identified as the main soluble factor in the TME responsible for suppressing IFN-α secretion by TA-pDCs through inhibition of IRF7 signaling (83, 84, 86). By exploring pan-cancer TCGA dataset, we found that immunosuppressive cytokines, such as TGF-β, PGE-2 and CSF1, are highly expressed across all cancer types, including melanoma samples (**Figure 1E**). Moreover, PTGES2 expression showed an inverse weak correlation with PDC signature and TGF-β signature (**Figures 1F, G**). On the contrary, IL-10 resulted expressed in a less relevant level. To further support this data, we tested the production of IL-10 and TGF-β, as well-known immunosuppressive cytokines in melanoma microenvironment, by melanoma cell lines. We found a significantly higher concentration of IL-10 (p = 0.0019) and TGF-β2 (p < 0.0001) protein in SN-mel146 compared to the others SN-mel (**Figures 6A, B**), while TGF-β1 protein was detected in all SN-mel (p = 0.0508) (**Figure 6C**). By RNAscope analysis of a set of PCM cases, we confirmed the expression of TGF-β in PCM area infiltrated by pDCs (**Figure 6D**), while the presence of IL-10 was almost undetectable (**Supplementary Figure S9**). Moreover, we detected a considerable inhibition of IRF7 constitutive expression in pDCs after their exposure to SN-mel (**Figure 6E**), providing evidence that TLR/MyD88-dependent signaling in pDCs was inhibited by melanoma secretome. In parallel, we explored STING-dependent signaling in pDCs stimulated with ADU S100 (**Figure 6F**). Activated STING recruits the TANK binding kinase-1 (TBK1) that is phosphorylated and in turn phosphorylates the interferon regulatory factor 3 (IRF3) and promotes its nuclear translocation (90) where IRF3 exerts its transcriptional function on immune-stimulated genes (ISG) and I-IFNs. By measuring the level of STING, TBK1 and IRF3 phosphorylation in pDCs exposed to melanoma supernatants, we could not detect evidence of molecular impairment of the STING pathway (**Figure 6F**). These results demonstrate that cGAS-STING signaling is not molecularly impaired in melanoma-conditioned pDCs; however, data using STING agonist confirmed a defective pDCs activation by melanoma-derived soluble factors (**Figures 4C, H**).

These data indicate that melanoma cells and the surrounding TME secrete immunosuppressive cytokines that might reduce the ability of PCM-associated pDCs to produce IRF7-dependent IFN-α, thus modulating pDCs anti-tumor function (91).

### Melanoma secretome impairs glycolysis in TLR7-activated pDCs

**Figure 7:**
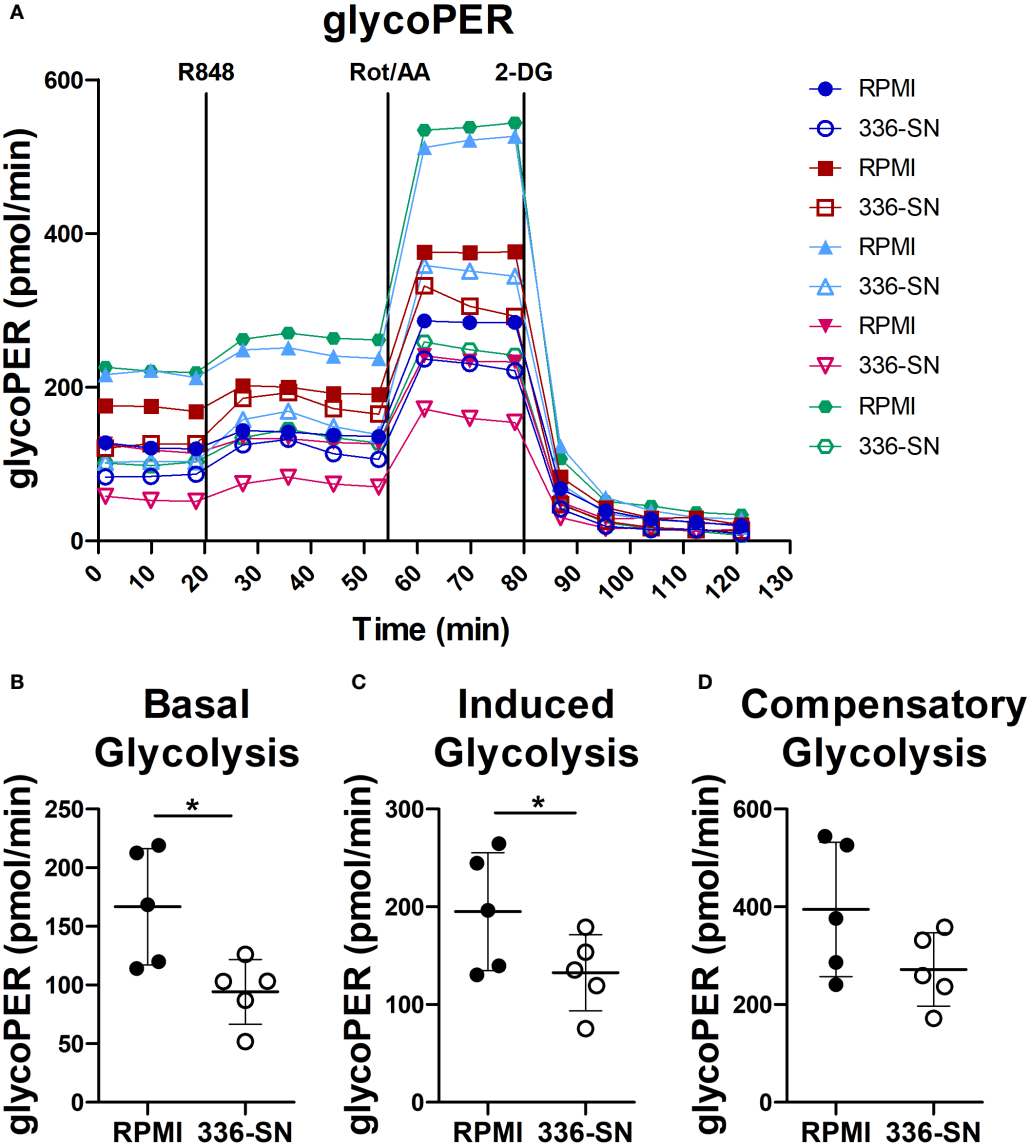
SN-mel exposure reduced glycolysis in pDCs. pDCs were cultured in RPMI medium or SN-mel 336 with no stimuli for 24h. pDCs were then subjected to the Glycolytic Rate Assay and glycolytic real-time proton efflux rate (glycoPER) is derived over the indicated time points **(A)**. Vertical lines indicate addition of R848, rotenone plus antimycin A (Rot/AA) and 2-deoxy-D-glucose (2-DG). Colors represent different healthy donors and dots represent the mean value of technical replicates. Basal glycolysis corresponds to the glycoPER value obtained at the time point before R848 injection **(B)**; p = 0.013). Induced glycolysis is calculated on the average glycoPER values over the four time points after R848 treatment **(C)**; p = 0.042). Compensatory glycolysis corresponds to glycoPER value measured at the third time point after Rot/AA injection **(D)**; p = 0.056). Bars represent the mean ± SD of biological replicates (n = 5). The statistical significance was calculated by two-tailed Paired Student’s t-test; * p < 0.05.

Administration of TLR-7/9 agonists promptly increases glycolysis in human pDCs, as measured by extracellular acidification rate (ECAR) (92, 93). Moreover, the inhibition of glycolysis by 2-deoxyglucose (2-DG) impairs type I IFN production and IFNA mRNA induction. Overall, these findings suggest that a metabolic switch towards glycolysis supports IFN-α production by activated pDCs (92, 93). Glucose deprivation and lactate accumulation, as we previously reported in SN-mel, might thus limit IFN-α production (49). We tested this hypothesis on freshly purified pDCs, by measuring extracellular acidification on the Seahorse XFe24 Extracellular Analyzer. The glycolytic proton efflux rate (glycoPER) increases upon R848 administration (**Figure 7A**). However, SN-mel336 conditioning hinders the basal glycolysis and, consequently, the R848-induced glycolysis in pDCs, as confirmed by a significant reduction in glycoPER (**Figures 7B, C**). GlycoPER highly correlates with extracellular lactate accumulation, the end product of aerobic glycolysis. These results suggest that SN-mel conditioned pDCs are impaired in glycolytic metabolism, independently of TLR7 activation. We could also observed a trend toward a reduced compensatory glycolysis following mitochondrial inhibition (i.e. oxidative phosphorylation inhibition by Rot/AA) in SN-mel336 exposed pDCs (**Figure 7D**), indicating a reduced ability of these cells to switch towards glycolysis to meet the cell energy demands.

Our results demonstrated that SN-mel negatively affects pDC glycolysis, suggesting a mechanism to explain the defective IFN-α production by SN-mel-exposed pDCs.

## Discussion

**Figure 8:**
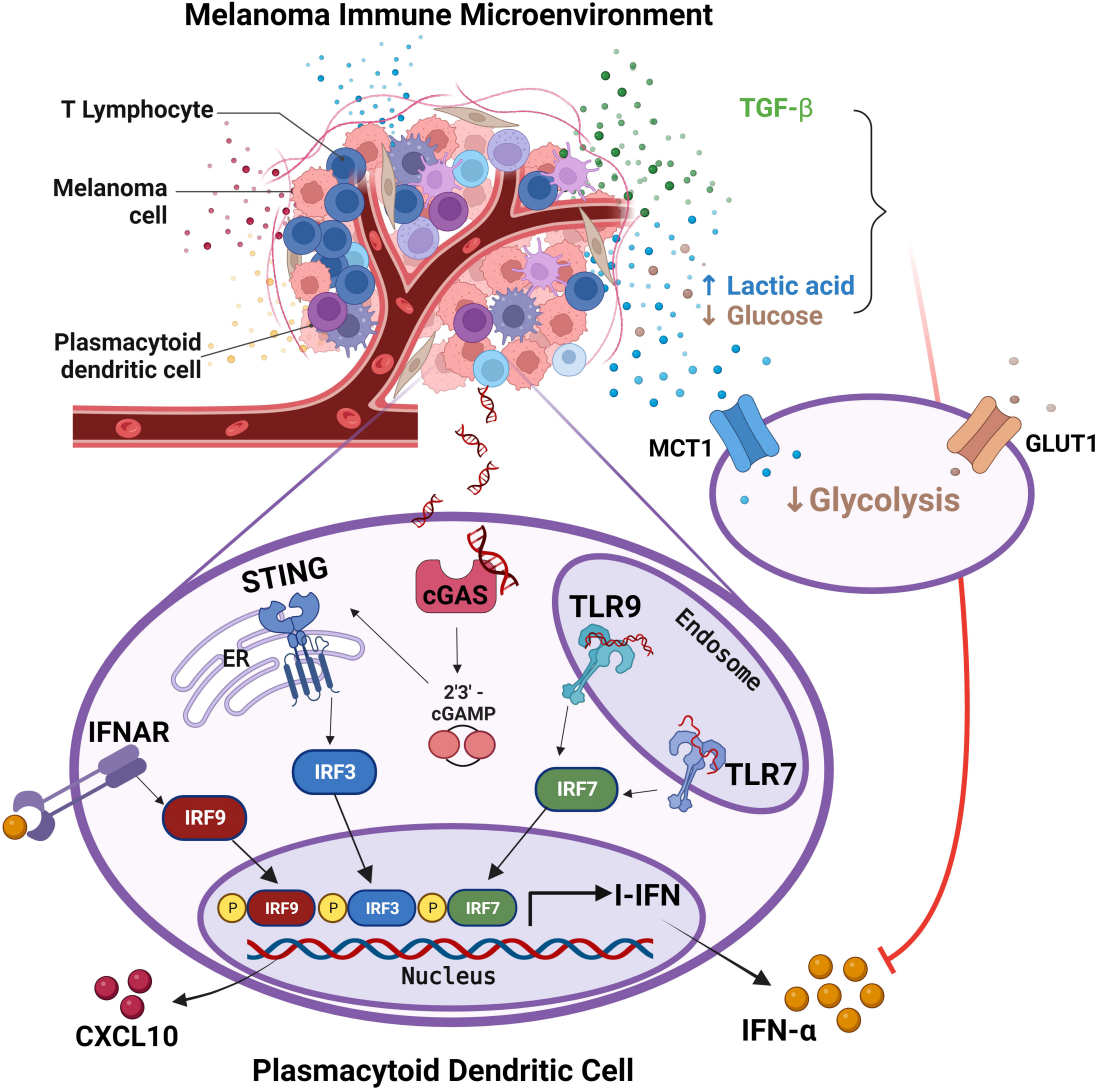
pDCs in melanoma immune microenvironment are impaired in IFN-α production. The graphical abstract summarize data obtained from the present study on melanoma-associated pDCs. Endogenous activation of pDCs results in I-IFN and CXCL10 production, with the latter contributing to the amplification of the local T-cell infiltration. Exposure of pDCs to melanoma TME hijacks pDC functions *via* TGF-β and oncometabolites (i.e. increased lactic acid and reduced glucose). Specifically, the reduction of both glucose uptake and lactic acid export by pDCs impairs their proficiency to IFN-α production. Adapted from “*Tumor Microenvironment*”, by BioRender.com (2023). Retrieved from https://app.biorender.com/biorender-templates.

PCM are divided in four major groups based on their molecular and immune landscape as well as their treatment options. Tumor immune microenvironment (TIME) in melanomas is highly complex in term of cell types, distribution and prognostic relevance (94). Our previous microscopic studies indicate that pDCs infiltrate early stages PCM, but they decrease over disease progression, and almost disappear in distant metastasis (46). Accordingly, circulating pDCs compartment collapsed in peripheral blood of CM patients with advanced disease stage, as a results of a defect of pDC differentiation from CD34^+^ progenitors. We and others could also detect a combined functional defect in circulating pDCs of melanoma patients in term of IFN-α and CXCL10 production, associated to poor survival (34, 46, 48, 49). Here, based on a set of *in vitro*, microscopic and *in silico* findings we found that pDCs infiltrating PCM are defective in IFN-α production and are hijacked in their functions, even when properly activated through the TLR7/9 or cGAS-STING signaling. Mechanistically, our findings suggest that PCM derived immunosuppressive cytokines as TGF-β and reduced glycolysis rewired fully differentiated pDCs towards a tolerogenic state (summarized by the graphical abstract in **Figure 8**).

Properly activated pDCs drive melanoma regression, as shown by experimental model and clinical studies (95–98). In PCM, pDC infiltration is heterogeneous, occurs early, and localizes at the invasive margin, the site where pDCs interact with CD8^+^ T cells (46, 99, 100). A set of data from the present study indicate that pDCs efficiency in I-IFN production is impaired, suggesting that they are not activated or are profoundly refractory to melanoma-derived stimuli. Based on RNA-Seq analysis, exposure to melanoma secretome rewired fully differentiated pDCs towards an IFN-defective and tolerogenic state. Specifically, transcripts for type I and type III interferons, T-cell recruiting pro-inflammatory cytokines (e.g., CXCL10) and co-stimulatory molecules (e.g., CD83, CD40) are significantly down-regulated. Moreover, melanoma secretome induces expression of a set of transcripts encoding for protein with tumor-promoting functions including CLEC17A, FR2, SMOX, TOX and ENPP1. Interestingly, TOX drives the T-cell exhaustion within TME, with decreased cytokine production and increased expression of several inhibitory surface receptors (76, 77, 101); ENPP1 hydrolyzes both extracellular ATP to AMP, which is metabolized to immune-suppressive adenosine, and 2′,3′-cGAMP playing a key role as negative regulator of the STING pathway (102–104).

Immunosuppressive cytokines, such as TGF-β, IL-10 and PGE-2, might play a role in promoting the pro-tumorigenic properties of pDCs (29, 83, 84, 105). Specifically, immunosuppressive cytokines inhibit secretion of IFN-α by TLR7/9-activated pDCs and involves inhibition of IRF7 expression in pDCs, thus reducing I-IFN production (83, 84, 87, 106–108). A variable level of IL-10, TGF-β1 and TGF-β2 has been measured in SN-mel. These findings were confirmed in the TCGA dataset and, for TGF-β1, on a small institutional cohort of pDC-enriched PCM. Accordingly, TAB1, a mediator of the TGF-β pathway, resulted as activated upstream regulator in pDCs exposed to SN-mel, suggesting that TGF-β might play a relevant role as IFN-α suppressor in pDCs.

The data presented in this study hint that pDCs impairment in melanoma might result from defective TLR/MyD88-dependent signaling (i.e., IRF7 activation) or from a broad perturbation of their metabolic functions (i.e., glycolysis). Metabolic pathways regulate innate and adaptive immune responses to activation signals, including TLR agonists (109). We previously demonstrated that lactic acidosis exerted immunosuppressive function on human pDCs (46, 49, 110). Lactic acidosis in the TME might be induced by high glycolytic tumor metabolism. Accordingly, RNA-Seq analysis revealed a glycolytic impairment in SN-mel exposed pDCs that has been confirmed by a reduced glycolytic extracellular acidification, suggesting that glucose deprivation or lactic acidosis by melanoma cells (49) hindered glycolytic metabolism in pDCs (31, 111) and consequently their innate immune functions, such as IFN-α secretion (92, 93). TLR stimulation caused metabolic reprogramming in DCs, which is critical for immune activation (112). Specifically, TLR-driven activation induces glycolysis to meet the energetic demands for immediate secretion of IFN-α by pDCs (92, 113). Moreover, TLR7/8 agonists up-regulates the oxidative phosphorylation and glutamine metabolism in pDCs, leading to a higher production of IFN-α and to increase T cell responses (114). On the contrary, inhibition of glutaminolysis and OXPHOS prevents pDC activation (114).

Among PRRs responsible for pDCs functions, the activation of STING pathway by tumor-derived dsDNA (61) plays an essential role in DC recognition of dying tumor cells and potently enhanced anti-tumor cytotoxic T cell responses (40, 59, 61, 115). By microscopy, using PLA, we found that endogenous activation of the cGAS-STING in PCM-infiltrating pDCs is limited to areas of microscopic regression. Longer DNA is more potent in activating cGAS by promoting liquid-like droplet formations in which cGAS and dsDNA are spatially concentrated for efficient cGAMP synthesis (116–118). The abundance of nucleases (DNases) in the extracellular milieu might explain the lack of dsDNA sensing via cGAS-STING by most pDCs (116). However, it is well known that cancer cells are often repleted with cytosolic dsDNA that might derive from the rupture of micronuclear envelopes as well as from acute genomic stressors induced by radiation, cisplatin, and intrinsic DNA damage (119). Moreover, cGAS cytosolic DNA sensors in cancer also include the mitochondrial DNA released as a consequence of oxidative stress and mitochondrial dysfunction (120). Finally, beyond the classical cGAS-cGAMP-STING axis, recent work has revealed that cGAS and STING may act independently from one another (121). Therefore, melanoma tumors lacking cGAS expression may still sustain active STING through other DNA binding proteins, such as IFI16 (121).

By stimulating blood purified pDCs with a STING agonist, we could detect pathway activation (i.e. STING, TBK1 and IRF3 phosphorylation), but reduced IFN-α production by SN-mel. In CM the expression and activation of components of the cGAS-STING pathway has been reported. Activation of STING signaling in human melanoma cell lines enhances their immunogenicity and susceptibility to lysis by tumor-infiltrating lymphocytes; conversely, defects in the STING signaling pathway protect melanoma cells from immune recognition by TILs and promote resistance to T cell–based immunotherapies (122, 123). Interestingly, the combination of cGAMP with radiation or blockade of PD-1/PD-L1 and CTLA-4 produces synergistic anti-tumor effects, which indicates that the cGAS–STING pathway is important for the sensing of cancer cells by the innate immune system (40, 61, 115) generating local and systemic anti-cancer immune responses (40, 42, 98, 124–130) and numerous clinical trials are ongoing (Clinicaltrials.gov study identifiers: NCT02675439; NCT03172936). The host response to type I IFNs bridges innate immunity to the adaptive immune response to tumor antigens (131). Specifically, type I IFNs-induced transcriptional signature is enriched in T cell-inflamed melanomas showing antitumor responses (132) and has been associated to ICB benefit (133, 134).

In summary, the present study demonstrates that the pDC compartment in PCM is defective in term of type I and III interferons production. Functional impairment of melanoma-conditioned pDCs is driven by immunosuppressive microenvironment and/or metabolic drift. In clinical perspective, the data obtained from the current work will enable to define better strategies for appropriate administration of STING and TLR agonists, in combination with existing or novel immunotherapies for CM. Of relevance, based on data from this study, major clinical benefits in term of survival, would derive from the treatment of early-stage PCM in the adjuvant setting, where the pDC compartment is still preserved. In general, the complete restoration of the pDC functions could be useful in melanoma’s treatment outcome. For instance, chemokine-based approaches (e.g. CXCL10) in combination with the standard BRAF and MEK inhibitors has been demonstrated to induce complete pathologic responses in mouse model (135). Among biomarkers for patient selection, responders are more likely to be identified within the CM subgroups lacking a TGF-β and IL-10 modulated TIME. It should be noted that a cross-regulatory mechanism between I-IFN signaling pathways has been identified in pDCs. Specifically, STING-mediated pathway induced expression of negative regulator SOCS1 that, through an autocrine inhibitory loop, dampened MyD88-dependent IFN production (14, 136) ruling out the combined administration of both TLR9 and STING agonists.

## Data availability statement

The data are publicly available at the following link: https://www.ncbi.nlm.nih.gov/geo/query/acc.cgi?acc=GSE234446.

## Ethics statement

The studies involving humans were approved by Institutional Review Board of ASST Spedali Civili of Brescia (IRB code: NP906). The studies were conducted in accordance with the local legislation and institutional requirements. Written informed consent for participation was not required from the participants or the participants’ legal guardians/next of kin in accordance with the national legislation and institutional requirements.

## Author contributions

MM, GF, AV, CT and WV contributed to conceiving and designing the study. MM, GF, VG, MO, VC, CT, AS, and LL collected and analysed the data. MM and GF carried out *in vitro* experiments. VG performed RNA-sequencing. MB, SZ and VC performed the histological examination. FM and MO performed the bioinformatics and statistical analysis. MM, GF, VC, MR, AS and LL developed methodology. MR provided facility. MM, GF, FC and WV wrote the manuscript. MR and WV supervised the study. All authors read and approved the submitted version of the manuscript.

## References

[1] Hodi FS O'Day SJ McDermott DF Weber RW Sosman JA Haanen JB. Improved survival with ipilimumab in patients with metastatic melanoma. N Engl J Med (2010) 363(8):711–23. doi: 10.1056/NEJMoa1003466 PMC3549297 20525992

[2] Wolchok JD Chiarion-Sileni V Gonzalez R Grob J-J Rutkowski P Lao CD. Long-term outcomes with nivolumab plus ipilimumab or nivolumab alone versus ipilimumab in patients with advanced melanoma. J Clin Oncol (2022) 40(2):127–37. doi: 10.1200/JCO.21.02229 PMC8718224 34818112

[3] Curti BD Faries MB. Recent advances in the treatment of melanoma. New Engl J Med (2021) 384(23):2229–40. doi: 10.1056/NEJMra2034861 34107182

[4] Eggermont A Blank C Mandalà M Long G Atkinson V Dalle S. Adjuvant pembrolizumab versus placebo in resected stage III melanoma (EORTC 1325-MG/KEYNOTE-054): distant metastasis-free survival results from a double-blind, randomised, controlled, phase 3 trial. Lancet Oncol (2021) 22(5):643–54. doi: 10.1016/S1470-2045(21)00065-6 33857412

[5] Larkin J Weber J Del Vecchio M Gogas H Arance A Dalle S. Adjuvant nivolumab versus ipilimumab (CheckMate 238 trial): Reassessment of 4-year efficacy outcomes in patients with stage III melanoma per AJCC-8 staging criteria. Eur J Cancer (2022) 173:285–96. doi: 10.1016/j.ejca.2022.06.041 35964471

[6] Luke J Rutkowski P Queirolo P Del Vecchio M Mackiewicz J Chiarion-Sileni V. Pembrolizumab versus placebo as adjuvant therapy in completely resected stage IIB or IIC melanoma (KEYNOTE-716): a randomised, double-blind, phase 3 trial. Lancet (2022) 399(10336):1718–29. doi: 10.1016/S0140-6736(22)00562-1 35367007

[7] Seth R Agarwala S Messersmith H Alluri K Ascierto P MB A. Systemic therapy for melanoma: ASCO guideline update. J Clin Oncol Off J Am Soc Clin Oncol (2023) 41(30):4794–820. doi: 10.1200/JCO.23.01136 37579248

[8] Collin M Bigley V. Human dendritic cell subsets: an update. Immunology. (2018) 154(1):3–20. doi: 10.1111/imm.12888 PMC5904714 29313948

[9] Reizis B. Plasmacytoid dendritic cells: development, regulation, and function. Immunity (2019) 50(1):37–50. doi: 10.1016/j.immuni.2018.12.027 PMC6342491 30650380

[10] Yin Z Dai J Deng J Sheikh F Natalia M Shih T. Type III IFNs are produced by and stimulate human plasmacytoid dendritic cells. J Immunol (2012) 189(6):2735–45. doi: 10.4049/jimmunol.1102038 PMC3579503 22891284

[11] Akira S Uematsu S Takeuchi O. Pathogen recognition and innate immunity. Cell. (2006) 124(4):783–801. doi: 10.1016/j.cell.2006.02.015 16497588

[12] Frittoli E Palamidessi A Iannelli F Zanardi F Villa S Barzaghi L. Tissue fluidification promotes a cGAS–STING cytosolic DNA response in invasive breast cancer. Nat Materials (2022) 22:644–655. doi: 10.1038/s41563-022-01431-x PMC10156599 36581770

[13] Bode C Fox M Tewary P Steinhagen A Ellerkmann RK Klinman D. Human plasmacytoid dentritic cells elicit a Type I Interferon response by sensing DNA via the cGAS-STING signaling pathway. Eur J Immunol (2016) 46(7):1615–21. doi: 10.1002/eji.201546113 PMC6389263 27125983

[14] Deb P Dai J Singh S Kalyoussef E Fitzgerald-Bocarsly P. Triggering of the cGAS–STING pathway in human plasmacytoid dendritic cells inhibits TLR9-mediated IFN production. J Immunol (2023) 205(1):223–36. doi: 10.4049/jimmunol.1800933 PMC7460725 32471881

[15] Hopfner K-P Hornung V. Molecular mechanisms and cellular functions of cGAS–STING signalling. Nat Rev Mol Cell Biol (2020) 21(9):501–21. doi: 10.1038/s41580-020-0244-x 32424334

[16] Medrano RFV Hunger A Mendonça SA Barbuto JAM Strauss BE. Immunomodulatory and antitumor effects of type I interferons and their application in cancer therapy. Oncotarget. (2017) 8(41):71249–84. doi: 10.18632/oncotarget.19531 PMC5642635 29050360

[17] Dunn GP Koebel CM Schreiber RD. Interferons, immunity and cancer immunoediting. Nat Rev Immunol (2006) 6(11):836–48. doi: 10.1038/nri1961 17063185

[18] Mcnab F Mayer-Barber K Sher A Wack A O'Garra A. Type I interferons in infectious disease. Nat Rev Immunol (2015) 15(2):87–103. doi: 10.1038/nri3787 25614319 PMC7162685

[19] Zitvogel L Galluzzi L Kepp O Smyth MJ Kroemer G. Type I interferons in anticancer immunity. Nat Rev Immunol (2015) 15(7):405–14. doi: 10.1038/nri3845 26027717

[20] Fuertes MB Woo S-R Burnett B Fu Y-X Gajewski TF. Type I interferon response and innate immune sensing of cancer. Trends Immunol (2013) 34(2):67–73. doi: 10.1016/j.it.2012.10.004 23122052 PMC3565059

[21] Swiecki M Colonna M. The multifaceted biology of plasmacytoid dendritic cells. Nat Rev Immunol (2015) 15(8):471–85. doi: 10.1038/nri3865 PMC4808588 26160613

[22] Swiecki M Colonna M. Unraveling the functions of plasmacytoid dendritic cells during viral infections, autoimmunity, and tolerance. Immunol Rev (2010) 234(1):142–62. doi: 10.1111/j.0105-2896.2009.00881.x PMC3507434 20193017

[23] Finotti G Tamassia N Cassatella M. Interferon-λs and plasmacytoid dendritic cells: A close relationship. Front Immunol (2017) 8. doi: 10.3389/fimmu.2017.01015 PMC5572322 28878776

[24] Harding SM Benci JL Irianto J Discher DE Minn AJ Greenberg RA. Mitotic progression following DNA damage enables pattern recognition within micronuclei. Nature. (2017) 548(7668):466–70. doi: 10.1038/nature23470 PMC5857357 28759889

[25] Chan Wah Hak C Rullan A Patin E Pedersen M Melcher A Harrington K. Enhancing anti-tumour innate immunity by targeting the DNA damage response and pattern recognition receptors in combination with radiotherapy. Front Oncol (2022) 12. doi: 10.3389/fonc.2022.971959 PMC9465159 36106115

[26] Luo R Onyshchenko K Wang L Gaedicke S Grosu A-L Firat E. Necroptosis-dependent immunogenicity of cisplatin: implications for enhancing the radiation-induced abscopal effect. Clin Cancer Res (2023) 29(3):667–83. doi: 10.1158/1078-0432.CCR-22-1591 36449659

[27] Zhang N Gao Y Huang Z Dai P Luo Y Wu Q. PARP inhibitor plus radiotherapy reshapes an inflamed tumor microenvironment that sensitizes small cell lung cancer to the anti-PD-1 immunotherapy. Cancer Lett (2022) 545. doi: 10.1016/j.canlet.2022.215852 35926817

[28] Demoulin S Herfs M Delvenne P Hubert P. Tumor microenvironment converts plasmacytoid dendritic cells into immunosuppressive/tolerogenic cells: insight into the molecular mechanisms. J Leukocyte Biol (2013) 93(3):343–52. doi: 10.1189/jlb.0812397 23136258

[29] Hartmann E Wollenberg B Rothenfusser S Wagner M Wellisch D Mack B. Identification and functional analysis of tumor-infiltrating plasmacytoid dendritic cells in head and neck cancer. Cancer Res (2003) 63(19):6478–87. doi: 10.1084/jem.20060401 14559840

[30] Koucky V Boucek J Fialova A. Immunology of plasmacytoid dendritic cells in solid tumors: A brief review. Cancers (Basel) (2019) 11(4):470. doi: 10.3390/cancers11040470 30987228 PMC6520684

[31] Raychaudhuri D Bhattacharya R Sinha BP Liu CSC Ghosh AR Rahaman O. Lactate induces pro-tumor reprogramming in intratumoral plasmacytoid dendritic cells. Front Immunol (2019) 10:1878. doi: 10.3389/fimmu.2019.01878 31440253 PMC6692712

[32] Guéry L Hugues S. Tolerogenic and activatory plasmacytoid dendritic cells in autoimmunity. Front Immunol (2013) 4. doi: 10.3389/fimmu.2013.00059 PMC3589693 23508732

[33] Ito T Yang M Wang YH Lande R Gregorio J Perng OA. Plasmacytoid dendritic cells prime IL-10-producing T regulatory cells by inducible costimulator ligand. J Exp Med (2007) 204(1):105–15. doi: 10.1084/jem.20061660 PMC2118437 17200410

[34] Aspord C Leccia MT Charles J Plumas J. Plasmacytoid dendritic cells support melanoma progression by promoting Th2 and regulatory immunity through OX40L and ICOSL. Cancer Immunol Res (2013) 1(6):402–15. doi: 10.1158/2326-6066 24778133

[35] Araújo EFD Medeiros DH Galdino NADL Condino-Neto A Calich VLG Loures FV. Tolerogenic plasmacytoid dendritic cells control paracoccidioides brasiliensis infection by inducting regulatory T cells in an IDO-dependent manner. PloS Pathogens (2016) 12(12):e1006115. doi: 10.1371/journal.ppat.1006115 27992577 PMC5215616

[36] Gehrie E van der Touw W Bromberg JS Ochando JC. Plasmacytoid dendritic cells in tolerance. Methods Mol Biol (2010) 677:127–47. doi: 10.1007/978-1-60761-869-0_9 PMC3721973 20941607

[37] Monti M Consoli F Vescovi R Bugatti M Vermi W. Human plasmacytoid dendritic cells and cutaneous melanoma. Cells. (2020) 9(2):417. doi: 10.3390/cells9020417 32054102 PMC7072514

[38] Mullins SR Vasilakos JP Deschler K Grigsby I Gillis P John J. Intratumoral immunotherapy with TLR7/8 agonist MEDI9197 modulates the tumor microenvironment leading to enhanced activity when combined with other immunotherapies. J ImmunoTherapy Cancer (2019) 7(1):244. doi: 10.1186/s40425-019-0724-8 PMC6739946 31511088

[39] Walshaw R Honeychurch J Choudhury A Illidge T. Toll-like receptor agonists and radiation therapy combinations: an untapped opportunity to induce anticancer immunity and improve tumor control. Int J Radiat oncology biology Phys (2020) 108(1):27–37. doi: 10.1016/j.ijrobp.2020.04.020 32339645

[40] Corrales L Glickman LH McWhirter SM Kanne DB Sivick KE Katibah GE. Direct activation of STING in the tumor microenvironment leads to potent and systemic tumor regression and immunity. Cell Rep (2015) 11(7):1018–30. doi: 10.1016/j.celrep.2015.04.031 PMC4440852 25959818

[41] Ramanjulu JM Pesiridis GS Yang J Concha N Singhaus R Zhang S-Y. Design of amidobenzimidazole STING receptor agonists with systemic activity. Nature. (2018) 564(7736):439–43. doi: 10.1038/s41586-018-0705-y 30405246

[42] Tel J Aarntzen EH Baba T Schreibelt G Schulte BM Benitez-Ribas D. Natural human plasmacytoid dendritic cells induce antigen-specific T-cell responses in melanoma patients. Cancer Res (2013) 73(3):1063–75. doi: 10.1158/0008-5472.CAN-12-2583 23345163

[43] Charles J Chaperot L Hannani D Bruder Costa J Templier I Trabelsi S. An innovative plasmacytoid dendritic cell line-based cancer vaccine primes and expands antitumor T-cells in melanoma patients in a first-in-human trial. OncoImmunology. (2020) 9(1):1738812. doi: 10.1080/2162402X.2020.1738812 32313721 PMC7153838

[44] Plumas J. Harnessing dendritic cells for innovative therapeutic cancer vaccines. Curr Opin Oncol (2022) 34(2):161–8. doi: 10.1097/CCO.0000000000000815 34930882

[45] Vermi W Bonecchi R Facchetti F Bianchi D Sozzani S Festa S. Recruitment of immature plasmacytoid dendritic cells (plasmacytoid monocytes) and myeloid dendritic cells in primary cutaneous melanomas. J Pathol (2003) 200(2):255–68. doi: 10.1002/path.1344 12754747

[46] Vescovi R Monti M Moratto D Paolini L Consoli F Benerini L. Collapse of the plasmacytoid dendritic cell compartment in advanced cutaneous melanomas by components of the tumor cell secretome. Cancer Immunol Res (2019) 7(1):12–28. doi: 10.1158/2326-6066.CIR-18-0141 30401679

[47] van den Hout M Koster BD Sluijter BJR Molenkamp BG van de Ven R van den Eertwegh AJM. Melanoma sequentially suppresses different DC subsets in the sentinel lymph node, affecting disease spread and recurrence. Cancer Immunol Res (2017) 5(11):969–77. doi: 10.1158/2326-6066.CIR-17-0110 28935649

[48] Failli A Legitimo A Orsini G Romanini A Consolini R. Numerical defect of circulating dendritic cell subsets and defective dendritic cell generation from monocytes of patients with advanced melanoma. Cancer Lett (2013) 337(2):184–92. doi: 10.1016/j.canlet.2013.05.013 23684927

[49] Monti M Vescovi R Consoli F Farina D Moratto D Berruti A. Plasmacytoid dendritic cell impairment in metastatic melanoma by lactic acidosis. Cancers. (2020) 12(8):2085. doi: 10.3390/cancers12082085 32731406 PMC7463681

[50] Aspord C Leccia MT Charles J Plumas J. Melanoma hijacks plasmacytoid dendritic cells to promote its own progression. Oncoimmunology. (2014) 3(1):e27402. doi: 10.4161/onci.27402 24701375 PMC3962506

[51] Lawrence M Huber W Pagès H Aboyoun P Carlson M Gentleman R. Software for computing and annotating genomic ranges. PloS Comput Biol (2013) 9(8):e1003118. doi: 10.1371/journal.pcbi.1003118 23950696 PMC3738458

[52] Love MI Huber W Anders S. Moderated estimation of fold change and dispersion for RNA-seq data with DESeq2. Genome Biol (2014) 15(12):1–21. doi: 10.1186/s13059-014-0550-8 PMC4302049 25516281

[53] Subramanian A Tamayo P Mootha VK Mukherjee S Ebert BL Gillette MA. Gene set enrichment analysis: A knowledge-based approach for interpreting genome-wide expression profiles. Proc Natl Acad Sci (2005) 102(43):15545–50. doi: 10.1073/pnas.0506580102 PMC1239896 16199517

[54] Mootha VK Lindgren CM Eriksson K-F Subramanian A Sihag S Lehar J. PGC-1α-responsive genes involved in oxidative phosphorylation are coordinately downregulated in human diabetes. Nat Genet (2003) 34(3):267–73. doi: 10.1038/ng1180 12808457

[55] Vivian J Rao AA Nothaft FA Ketchum C Armstrong J Novak A. Toil enables reproducible, open source, big biomedical data analyses. Nat Biotechnol (2017) 35(4):314–6. doi: 10.1038/nbt.3772 PMC5546205 28398314

[56] Hänzelmann S Castelo R Guinney J. GSVA: gene set variation analysis for microarray and RNA-Seq data. BMC Bioinf (2013) 14(1):7. doi: 10.1186/1471-2105-14-7 PMC3618321 23323831

[57] McInnes L Healy J Melville J. UMAP: uniform manifold approximation and projection for dimension reduction. (2020).

[58] Wenzel J Zahn S Bieber T Tüting T. Type I interferon-associated cytotoxic inflammation in cutaneous lupus erythematosus. Arch Dermatol Res (2009) 301(1):83–6. doi: 10.1007/s00403-008-0892-8 18784932

[59] Woo S-R Fuertes B M Corrales L Spranger S Furdyna J M Leung YK M. STING-dependent cytosolic DNA sensing mediates innate immune recognition of immunogenic tumors. Immunity. (2014) 41(5):830–42. doi: 10.1016/j.immuni.2014.10.017 PMC4384884 25517615

[60] Corrales L Gajewski TF. Endogenous and pharmacologic targeting of the STING pathway in cancer immunotherapy. Cytokine. (2016) 77:245–7. doi: 10.1016/j.cyto.2015.08.258 PMC4666728 26315534

[61] Deng L Liang H Xu M Yang X Burnette B Arina A. STING-dependent cytosolic DNA sensing promotes radiation-induced type I interferon-dependent antitumor immunity in immunogenic tumors. Immunity. (2014) 41(5):843–52. doi: 10.1016/j.immuni.2014.10.019 PMC5155593 25517616

[62] Kwon J Bakhoum S. The cytosolic DNA-sensing cGAS-STING pathway in cancer. Cancer Discovery (2020) 10(1):26–39. doi: 10.1158/2159-8290.CD-19-0761 PMC7151642 31852718

[63] Cai X Chiu YH Chen ZJ. The cGAS-cGAMP-STING pathway of cytosolic DNA sensing and signaling. Mol Cell (2014) 54(2):289–96. doi: 10.1016/j.molcel.2014.03.040 24766893

[64] Aivazian K. Regression in cutaneous melanoma: histological assessment, immune mechanisms and clinical implications. Pathology (2023) 55(2):227–35. doi: 10.1016/j.pathol.2022.11.005 36639333

[65] Cartron A Aldana P Khachemoune A. Reporting regression in primary cutaneous melanoma. Part 1: history, histological criteria and pathogenesis. Clin Exp Dermatol (2021) 46(1):28–33. doi: 10.1111/ced.14328 32597504

[66] Megjugorac NJ Young HA Amrute SB Olshalsky SL Fitzgerald-Bocarsly P. Virally stimulated plasmacytoid dendritic cells produce chemokines and induce migration of T and NK cells. J Leukoc Biol (2004) 75(3):504–14. doi: 10.1189/jlb.0603291 14742635

[67] Marsman C Lafouresse F Liao Y Baldwin TM Mielke LA Hu Y. Plasmacytoid dendritic cell heterogeneity is defined by CXCL10 expression following TLR7 stimulation. Immunol Cell Biol (2018) 96(10):1083–94. doi: 10.1111/imcb.12173 29870118

[68] Breiman A Robles MDL Trécesson S Echasserieau K Bernardeau K Drickamer K. Carcinoma-associated fucosylated antigens are markers of the epithelial state and can contribute to cell adhesion through CLEC17A (Prolectin). Oncotarget (2016) 7:14064–82. doi: 10.18632/oncotarget.7476 PMC4924698 26908442

[69] Graham SA Jégouzo SAF Yan S Powlesland AS Brady JP Taylor ME. Prolectin, a glycan-binding receptor on dividing B cells in germinal centers. J Biol Chem (2009) 284(27):18537–44. doi: 10.1074/jbc.M109.012807 PMC2709368 19419970

[70] Hwang YS Cho HJ Park ES Lim J Yoon HR Kim J-T. KLK6/PAR1 axis promotes tumor growth and metastasis by regulating cross-talk between tumor cells and macrophages. Cells. (2022) 11(24):4101. doi: 10.3390/cells11244101 36552865 PMC9777288

[71] Blackburn JS Liu I Coon CI Brinckerhoff CE. A matrix metalloproteinase-1/protease activated receptor-1 signaling axis promotes melanoma invasion and metastasis. Oncogene. (2009) 28(48):4237–48. doi: 10.1038/onc.2009.272 PMC2788659 19734937

[72] Zigler M Kamiya T Brantley E Villares G Bar-Eli M. PAR-1 and thrombin: the ties that bind the microenvironment to melanoma metastasis. Cancer Res (2011) 71(21):6561–66. doi: 10.1158/0008-5472.CAN-11-1432 PMC3206157 22009534

[73] Tellez C Bar-Eli M. Role and regulation of the thrombin receptor (PAR-1) in human melanoma. Oncogene. (2003) 22(20):3130–7. doi: 10.1038/sj.onc.1206453 12789289

[74] Kim S Kim D Roh S Hong I Kim H Ahn TS. Expression of spermine oxidase is associated with colorectal carcinogenesis and prognosis of patients. Biomedicines. (2022) 10(3):626. doi: 10.3390/biomedicines10030626 35327428 PMC8944969

[75] Liang C Zhao Y Chen C Huang S Deng T Zeng X. Higher TOX genes expression is associated with poor overall survival for patients with acute myeloid leukemia. Front Oncol (2021) 11. doi: 10.3389/fonc.2021.740642 PMC8532529 34692519

[76] Veldman J Rodrigues Plaça J Chong L Terpstra MM Mastik M Van Kempen LC. CD4+ T cells in classical Hodgkin lymphoma express exhaustion associated transcription factors TOX and TOX2. OncoImmunology (2022) 11(1). doi: 10.1080/2162402X.2022.2033433 PMC8803106 35111387

[77] Seo H Chen J González-Avalos E Samaniego-Castruita D Das A Wang Y. TOX and TOX2 transcription factors cooperate with NR4A transcription factors to impose CD8+ T cell exhaustion. Proc Natl Acad Sci United States America (2019) 116(25):12410–5. doi: 10.1073/pnas.1905675116 PMC6589758 31152140

[78] Seth S Oberdorfer L Hyde R Hoff K Thies V Worbs T. CCR7 essentially contributes to the homing of plasmacytoid dendritic cells to lymph nodes under steady-state as well as inflammatory conditions. J Immunol (2011) 186(6):3364–72. doi: 10.4049/jimmunol.1002598 21296980

[79] Ghanem M Shih A Khalili H Werth E Chakrabarty J Brown L. Proteomic and single-cell transcriptomic dissection of human plasmacytoid dendritic cell response to influenza virus. Front Immunol (2022) 13. doi: 10.3389/fimmu.2022.814627 PMC8984281 35401570

[80] Carenza C Calcaterra F Oriolo F Di Vito C Ubezio M Della Porta M. Costimulatory molecules and immune checkpoints are differentially expressed on different subsets of dendritic cells. Front Immunol (2019) 10. doi: 10.3389/fimmu.2019.01325 PMC6579930 31244860

[81] Aisenberg L Chattergoon M. Where do plasmacytoid dendritic cells find the energy? J leukocyte Biol (2021) 109(2):283–5. doi: 10.1002/JLB.4CE0820-271R PMC8500331 32876957

[82] Javelaud D Alexaki V-I Mauviel A. Transforming growth factor-β in cutaneous melanoma. Pigment Cell Melanoma Res (2008) 21(2):123–32. doi: 10.1111/j.1755-148X.2008.00450.x 18426405

[83] Sisirak V Vey N Goutagny N Renaudineau S Malfroy M Thys S. Breast cancer-derived transforming growth factor-beta and tumor necrosis factor-alpha compromise interferon-alpha production by tumor-associated plasmacytoid dendritic cells. Int J Cancer (2013) 133(3):771–8. doi: 10.1002/ijc.28072 23389942

[84] Bekeredjian-Ding I Schafer M Hartmann E Pries R Parcina M Schneider P. Tumour-derived prostaglandin E and transforming growth factor-beta synergize to inhibit plasmacytoid dendritic cell-derived interferon-alpha. Immunology. (2009) 128(3):439–50. doi: 10.1111/j.1365-2567.2009.03134.x PMC2770691 20067543

[85] Polak ME Borthwick NJ Gabriel FG Johnson P Higgins B Hurren J. Mechanisms of local immunosuppression in cutaneous melanoma. Br J Cancer (2007) 96(12):1879–87. doi: 10.1038/sj.bjc.6603763 PMC2359967 17565341

[86] Terra M Oberkampf M Fayolle C Rosenbaum P Guillerey C Dadaglio G. Tumor-derived TGFbeta alters the ability of plasmacytoid dendritic cells to respond to innate immune signaling. Cancer Res (2018) 78(11):3014–26. doi: 10.1158/0008-5472.CAN-17-2719 29523540

[87] Bruchhage KL Heinrichs S Wollenberg B Pries R. IL-10 in the microenvironment of HNSCC inhibits the CpG ODN induced IFN-alpha secretion of pDCs. Oncol Lett (2018) 15(3):3985–90. doi: 10.3892/ol.2018.7772 PMC5795883 29456743

[88] Sisirak V Faget J Gobert M Goutagny N Vey N Treilleux I. Impaired IFN-alpha production by plasmacytoid dendritic cells favors regulatory T-cell expansion that may contribute to breast cancer progression. Cancer Res (2012) 72(20):5188–97. doi: 10.1158/0008-5472.CAN-11-3468 22836755

[89] Labidi-Galy SI Sisirak V Meeus P Gobert M Treilleux I Bajard A. Quantitative and functional alterations of plasmacytoid dendritic cells contribute to immune tolerance in ovarian cancer. Cancer Res (2011) 71(16):5423–34. doi: 10.1158/0008-5472.CAN-11-0367 21697280

[90] Tanaka Y Chen Z. STING specifies IRF3 phosphorylation by TBK1 in the cytosolic DNA signaling pathway. Sci Signaling (2012) 5(214). doi: 10.1126/scisignal.2002521 PMC3549669 22394562

[91] Bencze D Fekete T Pázmándi K. Type I interferon production of plasmacytoid dendritic cells under control. Int J Mol Sci (2021) 22(8):4190. doi: 10.3390/ijms22084190 33919546 PMC8072550

[92] Bajwa G DeBerardinis R Shao B Hall B Farrar J Gill M. Cutting edge: critical role of glycolysis in human plasmacytoid dendritic cell antiviral responses. J Immunol (2016) 196(5):2004–9. doi: 10.4049/jimmunol.1501557 PMC4761472 26826244

[93] Saas P Varin A Perruche S Ceroi A. Recent insights into the implications of metabolism in plasmacytoid dendritic cell innate functions: Potential ways to control these functions. F1000Research. (2017) 6:456. doi: 10.12688/f1000research.11332.2 28580131 PMC5437952

[94] Ladányi A. Prognostic and predictive significance of immune cells infiltrating cutaneous melanoma. Pigment Cell melanoma Res (2015) 28(5):490–500. doi: 10.1111/pcmr.12371 25818762

[95] Drobits B Holcmann M Amberg N Swiecki M Grundtner R Hammer M. Imiquimod clears tumors in mice independent of adaptive immunity by converting pDCs into tumor-killing effector cells. J Clin Invest (2012) 122(2):575–85. doi: 10.1172/JCI61034 PMC3266798 22251703

[96] Stary G Bangert C Tauber M Strohal R Kopp T Stingl G. Tumoricidal activity of TLR7/8-activated inflammatory dendritic cells. J Exp Med (2007) 204(6):1441–51. doi: 10.1084/jem.20070021 PMC2118597 17535975

[97] Kalb ML Glaser A Stary G Koszik F Stingl G. TRAIL(+) human plasmacytoid dendritic cells kill tumor cells in *vitro*: mechanisms of imiquimod- and IFN-alpha-mediated antitumor reactivity. J Immunol (2012) 188(4):1583–91. doi: 10.4049/jimmunol.1102437 22231699

[98] Teulings HE Tjin EPM Willemsen KJ van der Kleij S Ter Meulen S Kemp EH. Anti-Melanoma immunity and local regression of cutaneous metastases in melanoma patients treated with monobenzone and imiquimod; a phase 2 a trial. Oncoimmunology. (2018) 7(4):e1419113. doi: 10.1080/2162402X.2017.1419113 29632737 PMC5889200

[99] Salio M Cella M Vermi W Facchetti F Palmowski MJ Smith CL. Plasmacytoid dendritic cells prime IFN-gamma-secreting melanoma-specific CD8 lymphocytes and are found in primary melanoma lesions. Eur J Immunol (2003) 33(4):1052–62. doi: 10.1002/eji.200323676 12672071

[100] Hernández SS Jakobsen MR Bak RO. Plasmacytoid dendritic cells as a novel cell-based cancer immunotherapy. Int J Mol Sci (2022) 23(19):11397. doi: 10.3390/ijms231911397 36232698 PMC9570010

[101] Blank CU Haining WN Held W Hogan PG Kallies A Lugli E. Defining ‘T cell exhaustion’. Nat Rev Immunol (2019) 19(11):665–74. doi: 10.1038/s41577-019-0221-9 PMC7286441 31570879

[102] Carozza JA Böhnert V Shaw KE Nguyen KC Skariah G Brown JA. 2’3’-cGAMP is an immunotransmitter produced by cancer cells and regulated by ENPP1. (2019). doi: 10.1101/539312

[103] Ding C Song Z Shen A Chen T Zhang A. Small molecules targeting the innate immune cGAS−STING−TBK1 signaling pathway. Acta Pharm Sin B (2020) 10(12):2272–98. doi: 10.1016/j.apsb.2020.03.001 PMC7745059 33354501

[104] Li L Yin Q Kuss P Maliga Z Millán JL Wu H. Hydrolysis of 2′3′-cGAMP by ENPP1 and design of nonhydrolyzable analogs. Nat Chem Biol (2014) 10(12):1043–8. doi: 10.1038/nchembio.1661 PMC4232468 25344812

[105] Hurwitz AA Watkins SK. Immune suppression in the tumor microenvironment: a role for dendritic cell-mediated tolerization of T cells. Cancer Immunology Immunother (2012) 61(2):289–93. doi: 10.1007/s00262-011-1181-5 PMC6948839 22237887

[106] Han N Zhang Z Jv H Hu J Ruan M Zhang C. Culture supernatants of oral cancer cells induce impaired IFN-alpha production of pDCs partly through the down-regulation of TLR-9 expression. Arch Oral Biol (2018) 93:141–8. doi: 10.1016/j.archoralbio.2018.06.006 29913322

[107] Combes A Camosseto V N’Guessan P Argüello RJ Mussard J Caux C. BAD-LAMP controls TLR9 trafficking and signalling in human plasmacytoid dendritic cells. Nat Commun (2017) 8(1):913. doi: 10.1038/s41467-017-00695-1 29030552 PMC5640662

[108] Fabricius D Neubauer M Mandel B Schütz C Viardot A V A. Prostaglandin E2 inhibits IFN-alpha secretion and Th1 costimulation by human plasmacytoid dendritic cells via E-prostanoid 2 and E-prostanoid 4 receptor engagement. J Immunol (2010) 184(2):677–84. doi: 10.4049/jimmunol.0902028 20018632

[109] Malinarich F Duan K Hamid R Bijin A Lin W Poidinger M. High mitochondrial respiration and glycolytic capacity represent a metabolic phenotype of human tolerogenic dendritic cells. J Immunol (Baltimore Md 1950) (2015) 194(11):5174–86. doi: 10.4049/jimmunol.1303316 25917094

[110] Gottfried E Kunz-Schughart L Ebner S Mueller-Klieser W Hoves S Andreesen R. Tumor-derived lactic acid modulates dendritic cell activation and antigen expression. Blood (2006) 107(5):2013–21. doi: 10.1182/blood-2005-05-1795 16278308

[111] Caronni N Simoncello F Stafetta F Guarnaccia C Ruiz-Moreno JS Opitz B. Downregulation of membrane trafficking proteins and lactate conditioning determine loss of dendritic cell function in lung cancer. Cancer Res (2018) 78(7):1685–99. doi: 10.1158/0008-5472.CAN-17-1307 29363545

[112] Joffre O Nolte M Spörri R Reis e Sousa C. Inflammatory signals in dendritic cell activation and the induction of adaptive immunity. Immunol Rev (2009) 227(1):234–47. doi: 10.1111/j.1600-065X.2008.00718.x 19120488

[113] Everts B Amiel E Huang SC-C Smith AM Chang C-H Lam WY. TLR-driven early glycolytic reprogramming via the kinases TBK1-IKKε supports the anabolic demands of dendritic cell activation. Nat Immunol (2014) 15(4):323–32. doi: 10.1038/ni.2833 PMC4358322 24562310

[114] Basit F Mathan T Sancho D de Vries I. Human dendritic cell subsets undergo distinct metabolic reprogramming for immune response. Front Immunol (2018) 9. doi: 10.3389/fimmu.2018.02489 PMC6230993 30455688

[115] Demaria O De Gassart A Coso S Gestermann N Di Domizio J Flatz L. STING activation of tumor endothelial cells initiates spontaneous and therapeutic antitumor immunity. Proc Natl Acad Sci (2015) 112(50):15408–13. doi: 10.1073/pnas.1512832112 PMC4687570 26607445

[116] A A C ZJ. cGAS in action: Expanding roles in immunity and inflammation. Science (2019) 363(6431). doi: 10.1126/science.aat8657 30846571

[117] Andreeva L Hiller B Kostrewa D Lässig C De Oliveira Mann CC Jan Drexler D. cGAS senses long and HMGB/TFAM-bound U-turn DNA by forming protein–DNA ladders. Nature. (2017) 549(7672):394–8. doi: 10.1038/nature23890 28902841

[118] Du M Chen Z. DNA-induced liquid phase condensation of cGAS activates innate immune signaling. Science (2018) 361(6403):704–9. doi: 10.1126/science.aat1022 PMC9417938 29976794

[119] Mackenzie KJ Carroll P Martin C-A Murina O Fluteau A Simpson DJ. cGAS surveillance of micronuclei links genome instability to innate immunity. Nature. (2017) 548(7668):461–5. doi: 10.1038/nature23449 PMC5870830 28738408

[120] Kitajima S Ivanova E Guo S Yoshida R Campisi M Sundararaman SK. Suppression of STING associated with LKB1 loss in KRAS-driven lung cancer. Cancer Discovery (2019) 9(1):34–45. doi: 10.1158/2159-8290.CD-18-0689 30297358 PMC6328329

[121] Dunphy G Flannery SM Almine JF Connolly DJ Paulus C Jønsson KL. Non-canonical activation of the DNA sensing adaptor STING by ATM and IFI16 mediates NF-κB signaling after nuclear DNA damage. Mol Cell (2018) 71(5):745–60.e5. doi: 10.1016/j.molcel.2018.07.034 30193098 PMC6127031

[122] Falahat R Perez-Villarroel P Mailloux AW Zhu G Pilon-Thomas S Barber GN. STING signaling in melanoma cells shapes antigenicity and can promote antitumor T-cell activity. Cancer Immunol Res (2019) 7(11):1837–48. doi: 10.1158/2326-6066.CIR-19-0229 PMC6825582 31462408

[123] Falahat R Berglund A Putney RM Perez-Villarroel P Aoyama S Pilon-Thomas S. Epigenetic reprogramming of tumor cell–intrinsic STING function sculpts antigenicity and T cell recognition of melanoma. Proc Natl Acad Sci (2021) 118(15):e2013598118. doi: 10.1073/pnas.2013598118 33827917 PMC8053941

[124] Pashenkov M Goess G Wagner C Hormann M Jandl T Moser A. Phase II trial of a toll-like receptor 9-activating oligonucleotide in patients with metastatic melanoma. J Clin Oncol (2006) 24(36):5716–24. doi: 10.1200/JCO.2006.07.9129 17179105

[125] Hofmann MA Kors C Audring H Walden P Sterry W Trefzer U. Phase 1 evaluation of intralesionally injected TLR9-agonist PF-3512676 in patients with basal cell carcinoma or metastatic melanoma. J Immunother (2008) 31(5):520–7. doi: 10.1097/CJI.0b013e318174a4df 18463532

[126] Aspord C Leccia MT Salameire D Laurin D Chaperot L Charles J. HLA-A(*)0201(+) plasmacytoid dendritic cells provide a cell-based immunotherapy for melanoma patients. J Invest Dermatol (2012) 132(10):2395–406. doi: 10.1038/jid.2012.152 22696054

[127] Atkins M Hodi F Thompson J McDermott D Hwu W Lawrence D. Pembrolizumab plus pegylated interferon alfa-2b or ipilimumab for advanced melanoma or renal cell carcinoma: dose-finding results from the phase ib KEYNOTE-029 study. Clin Cancer Res an Off J Am Assoc Cancer Res (2018) 24(8):1805–15. doi: 10.1158/1078-0432.CCR-17-3436 29358500

[128] Molenkamp B van Leeuwen P Meijer S Sluijter B Wijnands P Baars A. Intradermal CpG-B activates both plasmacytoid and myeloid dendritic cells in the sentinel lymph node of melanoma patients. Clin Cancer Res an Off J Am Assoc Cancer Res (2007) 13(10);2961–9. doi: 10.1158/1078-0432.CCR-07-0050 17504997

[129] Makowska Z Blumer T Duong F La Monica N Kandimalla E Heim M. Sequential induction of type I and II interferons mediates a long-lasting gene induction in the liver in response to a novel toll-like receptor 9 agonist. J Hepatol (2013) 58(4):743–9. doi: 10.1016/j.jhep.2012.11.038 23207140

[130] Wang S Campos J Gallotta M Gong M Crain C Naik E. Intratumoral injection of a CpG oligonucleotide reverts resistance to PD-1 blockade by expanding multifunctional CD8+ T cells. Proc Natl Acad Sci United States America (2016) 113(46):7240–9. doi: 10.1073/pnas.1608555113 PMC5135381 27799536

[131] Gajewski T Louahed J Brichard V. Gene signature in melanoma associated with clinical activity: a potential clue to unlock cancer immunotherapy. Cancer J (Sudbury Mass) (2010) 16(4):399–403. doi: 10.1097/PPO.0b013e3181eacbd8 20693853

[132] Harlin H Meng Y Peterson A Zha Y Tretiakova M S C. Chemokine expression in melanoma metastases associated with CD8+ T-cell recruitment. Cancer Res (2009) 69(7):3077–85. doi: 10.1158/0008-5472.CAN-08-2281 PMC3886718 19293190

[133] Jacquelot N Yamazaki T Roberti M Duong C A MC V L. Sustained Type I interferon signaling as a mechanism of resistance to PD-1 blockade. Cell Res (2019) 29(10):846–61. doi: 10.1038/s41422-019-0224-x PMC6796942 31481761

[134] Ye Z Dong H Li Y Ma T Huang H L HS. Prevalent homozygous deletions of type I interferon and defensin genes in human cancers associate with immunotherapy resistance. Clin Cancer Res an Off J Am Assoc Cancer Res (2018) 24(14):3299–308. doi: 10.1158/1078-0432.CCR-17-3008 PMC6050078 29618619

[135] Romano G Paradiso F Li P Shukla P Barger L El Naggar O. Microparticle-delivered cxcl9 prolongs braf inhibitor efficacy in melanoma. Cancer Immunol Res (2023) 11(5):558–69. doi: 10.1158/2326-6066.CIR-22-0224 PMC10159986 36820825

[136] Yu X Cai B Wang M Tan P Ding X Wu J. Cross-regulation of two type I interferon signaling pathways in plasmacytoid dendritic cells controls anti-malaria immunity and host mortality. Immunity. (2016) 45(5):1093–107. doi: 10.1016/j.immuni.2016.10.001 PMC7128466 27793594

