# Supplementary material for: Impaired activation of plasmacytoid dendritic cells via toll-like receptor 7/9 and STING is mediated by melanoma-derived immunosuppressive cytokines and metabolic drift
Source: Front Immunol. 2024 Jan 3;14:1227648. doi: 10.3389/fimmu.2023.1227648 (PMC10795195; doi:10.3389/fimmu.2023.1227648)
Supplement: Supplementary file 1 [file DataSheet_1.zip › Supplementary Tables.docx]

**Supplementary Data 1.** Gene lists of the six signature used for the Gene Set Variation Analysis (GSVA). The PDC signature was taken from our previous work (45) and I-IFNs/III-IFNs signature was created based on the known genes encoding for interferon-α, -β and –λ. The Interferon-α Response Signature and TGF-β Signature correspond to the Hallmark gene sets from the Molecular Signatures Database (MSigDB) collections. The Interleukin 10 Signaling and STING Signature were derived from the Reactome Canonical Pathway database (MSigDB).

**Supplementary Data 2.** GSEA-based functional enrichment analysis of SN-mel-exposed pDCs in comparison with RPMI-cultured pDCs revealed gene sets negatively and positively enriched. Functional gene sets are listed based on ES value. The functional gene sets related to innate immune response and I-IFN signaling are highlighted in light blue.

**Supplementary Figure S1.** Representative images of positive MUM1 and cGAS plasma cells in a PCM case. Magnification 400X. Scale bar 50 µm.

**Supplementary Figure S2.** (**A-B**) Representative photomicrographs showing dsDNA-cGAS interactions using the proximity ligation test (brown staining) combined with anti-E2.2 immunostaining (red staining) in PCM (**A**) and PCM with regression (**B**) cases. Magnification 400X. Scale bar 50 µm.

**Supplementary Figure S3. The amounts of IFN-α and CXCL10/IP-10 produced by pDCs are impaired by SN-mel.** pDCs isolated from HD were exposed to SN-mel for 24 h. pDCs were stimulated with R848 for 2 h (**D**) and 6 h (**F**) or with CpG-ODN 2216 for 6 h (**E, G**) as compared to unstimulated control samples (**A-C**). Brefeldin A was added 1 h (**A, D**) and 4 h (**B, E-G**) after stimuli. IFN-α (**A-E**) and CXCL10 (**F-G**) production is analysed by intracellular flow cytometry (**A-B, D-G**) and ELISA (**C**). Scatter dot plots show the percentage of IFN-α positive cells evaluated on BDCA-2^+^/CD123^+^ pDCs (**A-B**) or the Median of Fluorescence Intensity (MFI) evaluated on IFN-α^+^ or CXCL-10^+^ pDCs (**D-G**) or the IFN-α protein concentration *per* 1x10^5^ cells measured on pDC supernatants (**C**). Bars represent the mean of biological replicates. The statistical significance was calculated by One-way ANOVA (**A** p= 0.052; **B** p= 0.0002; **C** p= 0.0002; **D** p= 0.005; **E** p= 0.3; **F** p= 0.0002; **G** p= 0.01) and Bonferroni multiple comparison; * p < 0.05; ** p < 0.01; *** p < 0.001.

**Supplementary Figure S4. Quality Control (QC) of RNA-Seq data.** (**A**) Boxplots showing gene counts, normalized by sequencing depth, of each sample analyzed by RNAseq. (**B**) Heatmap of Euclidean samples distances based on normalized gene counts (rlog values) of samples analyzed by RNAseq.

**Supplementary Figure S5. Principal component analysis (PCA) run considering the whole gene expression on the SN-mel-exposed pDCs samples resting and R848 stimulated.** Pairs plots showing the combined projections of the first four components of the PCA in all analysed samples (**A**). The relevant features of the samples, such as donors (**B**), culture condition (**C-D**), and stimulation (**E**), are highlighted with different colour codes, as labelled.

**Supplementary Figure S6. Component loadings of the first three components of the PCA.** Plot showing the fifty most influencing loadings considering the first three PCs of the PC analysis.

**Supplementary Figure S7. Venn diagrams showing differentially expressed genes in SN-mel-exposed pDCs.** Venn diagrams show the number of DEGs in SN-mel146 and SN-mel336 exposed pDCs both resting (**A**) or stimulated with R848 (**B**). The sets of upregulated and downregulated genes are depicted in red and green colours respectively.

**Supplementary Figure S8. Gene Set Enrichment Analysis (GSEA) showing functional pathways enriched in SN-mel-exposed pDCs.** (**A**) Heatmap showing the normalized enrichment score (NES) values and related FDR q-values of pathways significantly modulated by SN-mel146 and/or SN-mel336 in pDC stimulated with R848, as identified by GSEA analysis. Enrichment plots representing a negative enrichment of the glycolysis gene set (**B**) and a positive enrichment of the TGF-β signalling (**C**) in SN-mel146 exposed pDCs compared to RPMI cultured pDCs.

**Supplementary Figure S9.** Representative images of TGF-β1 (upper panels) and IL-10 (lower panels) RNAscope in a PCM case. Magnification 400X. Scale bar 50 µm.

**Supplementary Table S1.** List of antibodies used for IHC and WB.

| **Immunohistochemistry** | | | | |
| --- | --- | --- | --- | --- |
| **Antibody** | **Clone** | **Isotype** | **Dilution** | **Producer** |
| **BDCA2** | 124B3.13 | Mouse IgG1 | 1:75 | Dendritics, Lyon, France |
| **MxA** | Mab M143 | Mouse IgG2a | 1:500 | Curtesy Dr.Otto A.Haller |
| **STING** | D2P2F | Rabbit | 1:25 | Cell Signaling Technology |
| **MART-1** | A103 | Mouse IgG1k | 1:100 | DAKO |
| **cGAS** | D1D3G | Rabbit | 1:100 | Cell Signaling Technology |
| **E2.2** | NCI-R159-6 | Rabbit | 1:500 | Abcam |
| **MUM1** | MUM1p | Mouse | Ready to use | DAKO |
| **Western Blot** | | | | |
| **p-STING** | D7C3S | Rabbit | 1:1000 | Cell Signaling Technology |
| **STING** | D2P2F | Rabbit | 1:1000 | Cell Signaling Technology |
| **TBK1** | D1B4 | Rabbit | 1:1000 | Cell Signaling Technology |
| **p-TBK1** | D52C2 [XP(R)] | Rabbit | 1:1000 | Cell Signaling Technology |
| **IRF3** | D83B9 | Rabbit | 1:1000 | Cell Signaling Technology |
| **p-IRF3** | 4D4G | Rabbit | 1:1000 | Cell Signaling Technology |
| **IRF7** | Polyclonal | Rabbit | 1:1000 | Cell Signaling Technology |
| **Histone H3** | Polyclonal | Rabbit | 1:5000 | Sigma-Aldrich |
| **α-tubulin** | DM1A | Mouse | 1:1000 | Sigma-Aldrich |

**Supplementary Table S2.** Samples distribution among the pan-cancer TCGA datasets.

|  | **Overall (N=2637)** |
| --- | --- |
| **Project_ID** |  |
| TCGA-BLCA | 405 (15.4%) |
| TCGA-COAD | 277 (10.5%) |
| TCGA-HNSC | 524 (19.9%) |
| TCGA-LUAD | 510 (19.3%) |
| TCGA-LUSC | 497 (18.8%) |
| TCGA-SKCM | 424 (16.1%) |
| **Stage_short_2** |  |
| Stage I | 672 (25.5%) |
| Stage II | 746 (28.3%) |
| Stage III | 656 (24.9%) |
| Stage IV | 557 (21.1%) |
| Missing | 6 (0.2%) |
| **Sample_type** |  |
| Metastatic | 327 (12.4%) |
| Primary Tumor | 2304 (87.4%) |
| Missing | 6 (0.2%) |
| **Sample_type_extended** |  |
| Distant Metastase | 142 (5.4%) |
| Lymph node | 191 (7.2%) |
| Primary Tumor | 2304 (87.4%) |

**Supplementary Table S3.** Signatures enrichment among primary tumor stages in the pan-cancer TCGA datasets.

|  | **TCGA-SKCM**  **(N=424)** | **TCGA-BLCA**  **(N=405)** | **TCGA-COAD**  **(N=277)** | | **TCGA-HNSC**  **(N=524)** | **TCGA-LUAD**  **(N=510)** | **TCGA-LUSC**  **(N=497)** | **p-value** |
| --- | --- | --- | --- | --- | --- | --- | --- | --- |
| **PDC signature** |  |  |  |  | |  |  |  |
| Mean (SD) | -0.253 (0.417) | -0.209 (0.364) | -0.0925 (0.386) | 0.0406 (0.436) | | 0.220 (0.357) | 0.0932 (0.391) | <0.001 |
| Median [Min, Max] | -0.382 [-0.866, 0.829] | -0.295 [-0.819, 0.809] | -0.137 [-0.726, 0.749] | 0.00529 [-0.731, 0.892] | | 0.294 [-0.753, 0.825] | 0.116 [-0.798, 0.829] |  |
| **I-IFNs and III-IFNs signature** |  |  |  |  | |  |  |  |
| Mean (SD) | -0.0681 (0.230) | 0.0475 (0.328) | -0.156 (0.237) | 0.224 (0.299) | | -0.0992 (0.282) | -0.0600 (0.302) | <0.001 |
| Median [Min, Max] | -0.0908 [-0.530, 0.542] | 0.00234 [-0.560, 0.843] | -0.205 [-0.558, 0.601] | 0.249 [-0.508, 0.847] | | -0.161 [-0.598, 0.826] | -0.130 [-0.598, 0.709] |  |
| **HALLMARK_INTERFERON ALPHA RESPONSE signature** |  |  |  |  | |  |  |  |
| Mean (SD) | -0.0812 (0.426) | -0.0640 (0.478) | -0.108 (0.377) | 0.240 (0.456) | | 0.0456 (0.394) | -0.173 (0.417) | <0.001 |
| Median [Min, Max] | -0.139 [-0.766, 0.751] | -0.192 [-0.772, 0.815] | -0.219 [-0.696, 0.753] | 0.388 [-0.699, 0.836] | | 0.0898 [-0.744, 0.743] | -0.303 [-0.766, 0.741] |  |
| **REACTOME_STING MEDIATED INDUCTION OF HOST IMMUNE RESPONSES signature** |  |  |  |  | |  |  |  |
| Mean (SD) | 0.0696 (0.257) | -0.0238 (0.279) | -0.0479 (0.243) | 0.127 (0.272) | | -0.174 (0.251) | -0.103 (0.252) | <0.001 |
| Median [Min, Max] | 0.0866 [-0.548, 0.633] | -0.0376 [-0.634, 0.640] | -0.0495 [-0.714, 0.586] | 0.154 [-0.648, 0.677] | | -0.197 [-0.699, 0.573] | -0.118 [-0.694, 0.561] |  |
| **HALLMARK_TGFB SIGNALING signature** |  |  |  |  | |  |  |  |
| Mean (SD) | -0.204 (0.193) | -0.0725 (0.256) | -0.137 (0.231) | 0.182 (0.293) | | 0.0617 (0.263) | -0.0318 (0.281) | <0.001 |
| Median [Min, Max] | -0.225 [-0.598, 0.549] | -0.0892 [-0.590, 0.628] | -0.169 [-0.572, 0.642] | 0.238 [-0.601, 0.661] | | 0.0748 [-0.600, 0.586] | -0.0312 [-0.595, 0.640] |  |
| **REACTOME_INTERLEUKIN 10 SIGNALING signature** |  |  |  |  | |  |  |  |
| Mean (SD) | -0.274 (0.379) | -0.179 (0.450) | -0.144 (0.405) | 0.191 (0.368) | | 0.208 (0.358) | 0.0734 (0.435) | <0.001 |
| Median [Min, Max] | -0.383 [-0.819, 0.666] | -0.316 [-0.826, 0.746] | -0.185 [-0.731, 0.707] | 0.248 [-0.683, 0.759] | | 0.302 [-0.678, 0.760] | 0.153 [-0.849, 0.798] |  |

**Supplementary Table S4.** Signatures enrichment among primary tumor stages in the pan-cancer TCGA datasets.

|  | **Stage I**  **(N=595)** | **Stage II**  **(N=670)** | **Stage III**  **(N=510)** | **Stage IV**  **(N=529)** | **p-value** |
| --- | --- | --- | --- | --- | --- |
| **PDC signature** |  |  |  |  |  |
| Mean (SD) | 0.172 (0.390) | -0.0515 (0.427) | -0.0247 (0.424) | -0.0530 (0.411) | <0.001 |
| Median [Min, Max] | 0.227 [-0.788, 0.829] | -0.115 [-0.840, 0.825] | -0.0383 [-0.808, 0.892] | -0.106 [-0.819, 0.862] |  |
| **I-IFNs and III-IFNs signature** |  |  |  |  |  |
| Mean (SD) | -0.0717 (0.300) | -0.0195 (0.312) | -0.0225 (0.321) | 0.138 (0.315) | <0.001 |
| Median [Min, Max] | -0.138 [-0.598, 0.725] | -0.0849 [-0.598, 0.843] | -0.0772 [-0.563, 0.826] | 0.144 [-0.560, 0.847] |  |
| **HALLMARK_INTERFERON ALPHA RESPONSE signature** |  |  |  |  |  |
| Mean (SD) | -0.0368 (0.427) | -0.0189 (0.451) | -0.0220 (0.461) | 0.0776 (0.470) | <0.001 |
| Median [Min, Max] | -0.0824 [-0.751, 0.823] | -0.0580 [-0.766, 0.836] | -0.108 [-0.754, 0.808] | 0.143 [-0.772, 0.825] |  |
| **REACTOME_STING MEDIATED INDUCTION OF HOST IMMUNE RESPONSES signature** |  |  |  |  |  |
| Mean (SD) | -0.119 (0.264) | -0.0228 (0.270) | -0.0457 (0.285) | 0.0387 (0.288) | <0.001 |
| Median [Min, Max] | -0.137 [-0.699, 0.632] | -0.0367 [-0.644, 0.646] | -0.0555 [-0.714, 0.612] | 0.0403 [-0.694, 0.677] |  |
| **HALLMARK_TGFB SIGNALING signature** |  |  |  |  |  |
| Mean (SD) | 0.00977 (0.276) | -0.0438 (0.290) | 0.000746 (0.282) | 0.0828 (0.301) | <0.001 |
| Median [Min, Max] | 0.00171 [-0.600, 0.640] | -0.0756 [-0.596, 0.612] | -0.0264 [-0.589, 0.644] | 0.0947 [-0.601, 0.661] |  |
| **REACTOME_INTERLEUKIN 10 SIGNALING signature** |  |  |  |  |  |
| Mean (SD) | 0.140 (0.406) | -0.0130 (0.448) | 0.0127 (0.449) | 0.0330 (0.423) | <0.001 |
| Median [Min, Max] | 0.241 [-0.745, 0.760] | -0.00774 [-0.826, 0.798] | 0.0530 [-0.849, 0.751] | 0.0634 [-0.818, 0.759] |  |

**Supplementary Table S5.** **Component loadings of the first three components of the PCA.** Table showing the fifty most influencing genes considering the first three PCs of the PC analysis.

| **TOP50 PC Loadings** | | | | | | **BOTTOM50 PC Loadings** | | | | | |
| --- | --- | --- | --- | --- | --- | --- | --- | --- | --- | --- | --- |
| **PC1** | | **PC2** | | **PC3** | | **PC1** | | **PC2** | | **PC3** | |
| **HGNC** | **Value** | **HGNC** | **Value** | **HGNC** | **Value** | **HGNC** | **Value** | **HGNC** | **Value** | **HGNC** | **Value** |
| **IFNW1** | 0.065217 | **XIST** | 0.047106 | **SLC2A5** | 0.055353 | **DUSP7** | -0.03585 | **RPS4Y1** | -0.05121 | **EIF1AY** | -0.04662 |
| **IFNA8** | 0.065068 | **KRT17** | 0.045885 | **MRC2** | 0.047726 | **KCNA5** | -0.0337 | **SIGLEC17P** | -0.04892 | **KDR** | -0.04463 |
| **IFNA2** | 0.064902 | **KRT16** | 0.036195 | **ECEL1** | 0.042586 | **RPP25** | -0.0274 | **SLC2A5** | -0.04678 | **CRIP2** | -0.04095 |
| **IFNB1** | 0.064796 | **HLA-B** | 0.034767 | **FCRL4** | 0.041118 | **SNAI3** | -0.02701 | **DDX3Y** | -0.04639 | **HLA-DQB1** | -0.04049 |
| **IFNA17** | 0.064538 | **MNDA** | 0.034555 | **IFITM10** | 0.04055 | **LRRC25** | -0.02513 | **USP9Y** | -0.04622 | **MMP12** | -0.03884 |
| **IFNA14** | 0.064484 | **MYBL2** | 0.033287 | **ENPP1** | 0.040144 | **C15orf39** | -0.02366 | **KDM5D** | -0.0454 | **AK4** | -0.03873 |
| **IFNA6** | 0.064369 | **CTTN** | 0.032761 | **XIST** | 0.039501 | **B4GAT1** | -0.02327 | **ZFY** | -0.04345 | **HILPDA** | -0.03815 |
| **IFNA4** | 0.063733 | **IFI27** | 0.032269 | **LINC00472** | 0.038294 | **HHEX** | -0.02266 | **PADI2** | -0.04259 | **KRT17P6** | -0.03792 |
| **IFNA21** | 0.063562 | **HLA-DRB6** | 0.031983 | **ACY3** | 0.037732 | **IRF2BPL** | -0.02237 | **CXCL5** | -0.04249 | **AJAP1** | -0.03707 |
| **IFNA7** | 0.063501 | **P2RX2** | 0.031903 | **ASB2** | 0.035413 | **POLR1G** | -0.02216 | **TXLNGY** | -0.04223 | **PDK1** | -0.0369 |
| **IFNA10** | 0.063219 | **SLC32A1** | 0.030884 | **LGR6** | 0.035396 | **RNF166** | -0.02158 | **HLA-DRA** | -0.0413 | **DDX3Y** | -0.03682 |
| **IFNA13** | 0.063208 | **HLA-DPA1** | 0.030172 | **SLC37A2** | 0.035121 | **ADAMTS4** | -0.02131 | **EIF1AY** | -0.04085 | **RPS4Y1** | -0.03649 |
| **IFNA16** | 0.06318 | **MMP12** | 0.029601 | **NLRP6** | 0.032645 | **KBTBD7** | -0.02117 | **IFITM10** | -0.04059 | **CX3CR1** | -0.03585 |
| **IFNA5** | 0.063149 | **C4BPB** | 0.028959 | **WDR64** | 0.032191 | **DUSP6** | -0.02109 | **LRRC15** | -0.03915 | **ADM** | -0.0354 |
| **IFNL3** | 0.062478 | **TUBB** | 0.028732 | **RHBDL3** | 0.032037 | **ARL11** | -0.0209 | **SCARA5** | -0.03808 | **KDM5D** | -0.03482 |
| **IFNL2** | 0.062463 | **TAP2** | 0.028005 | **IQCD** | 0.031843 | **KAT14** | -0.02079 | **TOX2** | -0.03777 | **TXLNGY** | -0.03464 |
| **IFNL1** | 0.061717 | **CCL19** | 0.027984 | **TAP2** | 0.031725 | **HERPUD1** | -0.02073 | **MRC2** | -0.03646 | **LTA** | -0.03451 |
| **IFNA1** | 0.061367 | **NES** | 0.027794 | **SFRP5** | 0.031514 | **FAM217B** | -0.02035 | **IQCD** | -0.0353 | **LPA** | -0.03445 |
| **TAL1** | 0.060643 | **ANKRD1** | 0.027271 | **TACSTD2** | 0.03138 | **MARCHF1** | -0.02028 | **UTY** | -0.03529 | **FNDC4** | -0.03438 |
| **IL12A** | 0.060227 | **COBL** | 0.026877 | **JAG2** | 0.0313 | **KLHL33** | -0.01986 | **SMOX** | -0.03499 | **USP9Y** | -0.03434 |
| **IFNWP9** | 0.05954 | **LGALS2** | 0.026633 | **SPNS3** | 0.030989 | **PLAU** | -0.01981 | **ENPP1** | -0.03466 | **KRT17** | -0.03433 |
| **IFNWP18** | 0.05897 | **ACKR1** | 0.026116 | **CD1D** | 0.03074 | **PROC** | -0.01978 | **BMP8B** | -0.03402 | **HLA-DRA** | -0.03363 |
| **IFNE** | 0.058243 | **BARX1** | 0.025825 | **IL1RL2** | 0.03033 | **BAIAP2-DT** | -0.01909 | **LTA** | -0.03401 | **DLGAP3** | -0.03196 |
| **FOXA1** | 0.056311 | **KHK** | 0.025813 | **HLA-DPA1** | 0.030212 | **PLA2G15** | -0.019 | **NCMAP** | -0.0334 | **SLC29A1** | -0.0319 |
| **IFNWP5** | 0.055913 | **SSTR3** | 0.025581 | **CDO1** | 0.029827 | **ORAI3** | -0.0189 | **ECEL1** | -0.03338 | **GPNMB** | -0.03183 |
| **PTGS2** | 0.055273 | **GGT2P** | 0.024438 | **QPCT** | 0.029782 | **SLC20A1** | -0.01878 | **APLN** | -0.03299 | **RGS7** | -0.03152 |
| **POU3F1** | 0.053603 | **KDR** | 0.024176 | **KIT** | 0.029738 | **MCAT** | -0.01867 | **IGLON5** | -0.03295 | **MIR210HG** | -0.03114 |
| **AFAP1** | 0.052945 | **LINC00472** | 0.02408 | **SMOX** | 0.029707 | **SNX18** | -0.01847 | **GIPR** | -0.03274 | **RPS7P6** | -0.0311 |
| **FST** | 0.052157 | **HBA1** | 0.023488 | **TGM2** | 0.029519 | **SERPINF2** | -0.0184 | **BTBD11** | -0.03244 | **P4HA2** | -0.03078 |
| **CCL3L1** | 0.051832 | **IFNNP1** | 0.023098 | **SIGLEC17P** | 0.029473 | **TRIM65** | -0.01839 | **GRB7** | -0.03198 | **KIAA1549L** | -0.03055 |
| **MYLK3** | 0.051677 | **HCAR1** | 0.02291 | **CLEC17A** | 0.029256 | **LONRF2** | -0.01818 | **XPNPEP2** | -0.03197 | **RAMP2-AS1** | -0.03033 |
| **NEU4** | 0.051318 | **CLLU1-AS1** | 0.022406 | **TGM5** | 0.029133 | **SKP2** | -0.01807 | **CDH3** | -0.03197 | **PLEKHD1** | -0.03015 |
| **RANBP3L** | 0.051174 | **CX3CR1** | 0.021834 | **CD14** | 0.028846 | **CIPC** | -0.01787 | **F2R** | -0.0319 | **SLC32A1** | -0.02947 |
| **CCL4** | 0.050771 | **PNOC** | 0.02142 | **OXCT2** | 0.028521 | **TREML2** | -0.01781 | **CLEC17A** | -0.03177 | **SGSM1** | -0.02925 |
| **CCL4L2** | 0.049051 | **MAP2K6** | 0.021133 | **CDH17** | 0.028508 | **INAFM2** | -0.01778 | **HLA-DQB1** | -0.03149 | **HLA-DQA1** | -0.02883 |
| **IL6** | 0.049042 | **HBA2** | 0.021 | **NCMAP** | 0.028465 | **SEPHS2** | -0.01772 | **HNF1B** | -0.03125 | **CTTN** | -0.02849 |
| **GCSAML** | 0.048447 | **CCND2** | 0.020758 | **RASD1** | 0.028387 | **MGAT3** | -0.01766 | **PLXDC2** | -0.03098 | **LINC01619** | -0.02832 |
| **DKK1** | 0.047909 | **EPHB1** | 0.020572 | **IGLON5** | 0.028355 | **RAB11FIP4** | -0.01762 | **CHST15** | -0.0305 | **BNIP3** | -0.0283 |
| **FOXC1** | 0.047411 | **PAGE2B** | 0.020534 | **F2R** | 0.028327 | **DISP1** | -0.01762 | **ASB2** | -0.03049 | **NGFR** | -0.02817 |
| **CCDC83** | 0.047096 | **OAF** | 0.020533 | **FRAT2** | 0.028261 | **WDR81** | -0.01727 | **SLC37A2** | -0.03042 | **PCDH15** | -0.02814 |
| **PLEKHA4** | 0.047033 | **RAMP2** | 0.02046 | **CD101** | 0.027854 | **CHST10** | -0.01725 | **MAL** | -0.0303 | **PFKFB4** | -0.02808 |
| **SLC1A2** | 0.046884 | **C5orf64** | 0.020422 | **OXCT2P1** | 0.027718 | **SH2D3C** | -0.0172 | **TESC** | -0.03025 | **TRPV6** | -0.028 |
| **MSGN1** | 0.046195 | **LILRA4** | 0.020406 | **IGHV3-73** | 0.027685 | **BCL7A** | -0.0171 | **ARHGDIG** | -0.03016 | **CCL19** | -0.02771 |
| **CCL3** | 0.046136 | **RAMP2-AS1** | 0.020284 | **TOX2** | 0.027372 | **TLR10** | -0.01699 | **SMIM24** | -0.03011 | **CLLU1** | -0.0274 |
| **NSRP1P1** | 0.045236 | **FXYD6** | 0.019984 | **GPR17** | 0.027259 | **SERTAD2** | -0.01697 | **WDR64** | -0.0301 | **ANKRD20A8P** | -0.02731 |
| **TWIST1** | 0.04488 | **SLC29A1** | 0.019915 | **ZNF516** | 0.027204 | **SETD7** | -0.01693 | **MUC12** | -0.03007 | **COL6A2** | -0.02729 |
| **IL27** | 0.044747 | **RGS7** | 0.019838 | **RHOBTB1** | 0.027049 | **CALHM2** | -0.01688 | **CD300LB** | -0.02958 | **WNT10A** | -0.02728 |
| **UNC5C** | 0.044676 | **WNT10A** | 0.019804 | **PALD1** | 0.026835 | **CISH** | -0.01686 | **ITM2A** | -0.02933 | **HPD** | -0.02724 |
| **NR5A2** | 0.04458 | **DLGAP3** | 0.019759 | **IPCEF1** | 0.026788 | **RNF125** | -0.01682 | **AMZ1** | -0.02933 | **DHRS9** | -0.02704 |
| **CCL5** | 0.044566 | **IL32** | 0.019412 | **PRMT9** | 0.026714 | **PIP5KL1** | -0.01671 | **TMEM273** | -0.0291 | **TLE1** | -0.02694 |

**Supplementary Table S6.** List of up-regulated genes resulting from the gene enrichment and functional annotation analysis in R848-stimulated pDCs.

| **HGNC symbol** | **R848 vs CTRL** | |
| --- | --- | --- |
|  | **Log_2_FC** | **p adj** |
| **IFNA1** | 8,46 | 3.22E-24 |
| **IFNL2** | 10,63 | 6.86E-86 |
| **IFNL3** | 9,44 | 1.63E-49 |
| **IFNL1** | 8,56 | 1.28E-26 |
| **CXCL2** | 8,52 | 1.65E-38 |
| **IFNA10** | 8,39 | 1.29E-24 |
| **IFNA6** | 7,94 | 1.02E-16 |
| **CCL5** | 7,86 | 7.10E-113 |
| **IFNA2** | 7,64 | 2.43E-15 |
| **IFNA16** | 7,64 | 1.30E-16 |
| **IFNB1** | 7,56 | 7.19E-15 |
| **IFNA14** | 7,3 | 2.30E-13 |
| **CCL4** | 7,28 | 6.37E-79 |
| **IFNW1** | 7,26 | 4.82E-13 |
| **IFNA8** | 6,92 | 1.38E-11 |
| **CCL3** | 6,91 | 3.28E-59 |
| **IFNA5** | 6,82 | 3.69E-11 |
| **IFNA17** | 6,77 | 1.20E-10 |
| **IFNA7** | 6,68 | 1.24E-10 |
| **IFNA13** | 6,6 | 2.46E-10 |
| **IFNA4** | 6,57 | 4.45E-10 |
| **IFNE** | 6,47 | 1.47E-10 |
| **IFNA21** | 5,98 | 5.43E-08 |
| **TNFSF15** | 5,66 | 1.49E-61 |
| **IFIT2** | 5,5 | 7.54E-55 |
| **LILRB2** | 4,98 | 2.89E-108 |
| **IL18RAP** | 4,93 | 7.65E-95 |
| **CXCL-8** | 4,54 | 1.10E-77 |
| **CXCL3** | 4,41 | 1.20E-22 |
| **CXCL-11** | 4,392 | 3.97E-13 |
| **TNFSF9** | 4,19 | 0.0001 |
| **CXCL-1** | 4,18 | 1.98E-26 |
| **TNFAIP6** | 4,17 | 1.15E-12 |
| **CD70** | 3,98 | 6.17E-47 |
| **TNFSF14** | 3,79 | 4.62E-17 |
| **CXCL-10** | 3,692 | 3.66E-06 |
|  | **Log_2_FC** | **p adj** |
| **LILRA5** | 3,69 | 4.85E-41 |
| **CXCL-9** | 3,625 | 7.11E-06 |
| **CD40** | 3,62 | 8.48E-52 |
| **IFIT3** | 3,48 | 9.41E-17 |
| **CCR7** | 3,41 | 1.79E-36 |
| **TNFSF4** | 3,16 | 1.61E-27 |
| **TNFAIP8** | 2,96 | 2.69E-61 |
| **TNFRSF10B** | 2,82 | 3.11E-16 |
| **CD83** | 2,81 | 4.23E-23 |
| **IFNL4** | 2,74 | 0.037 |
| **IRF2** | 2,73 | 7.74E-48 |
| **IFIT1** | 2,44 | 7.33E-06 |
| **TNFSF10** | 2,19 | 2.55E-08 |
| **IRF4** | 1,94 | 1.33E-19 |
| **IFIT5** | 1,93 | 2.93E-11 |
| **TNFSF18** | 1,88 | 2.12E-10 |
| **CD80** | 1,82 | 6.66E-27 |
| **ISG20** | 1,53 | 4.78E-07 |
| **TNFRSF9** | 1,41 | 0.00011 |
| **ISG15** | 1,35 | 0.00029 |
| **TNFRSF11A** | 1,23 | 9.07E-07 |
| **CD86** | 1,03 | 2.46E-07 |
