# Supplementary figures and images for: Impaired activation of plasmacytoid dendritic cells via toll-like receptor 7/9 and STING is mediated by melanoma-derived immunosuppressive cytokines and metabolic drift
Source: Front Immunol. 2024 Jan 3;14:1227648. doi: 10.3389/fimmu.2023.1227648 (PMC10795195; doi:10.3389/fimmu.2023.1227648)

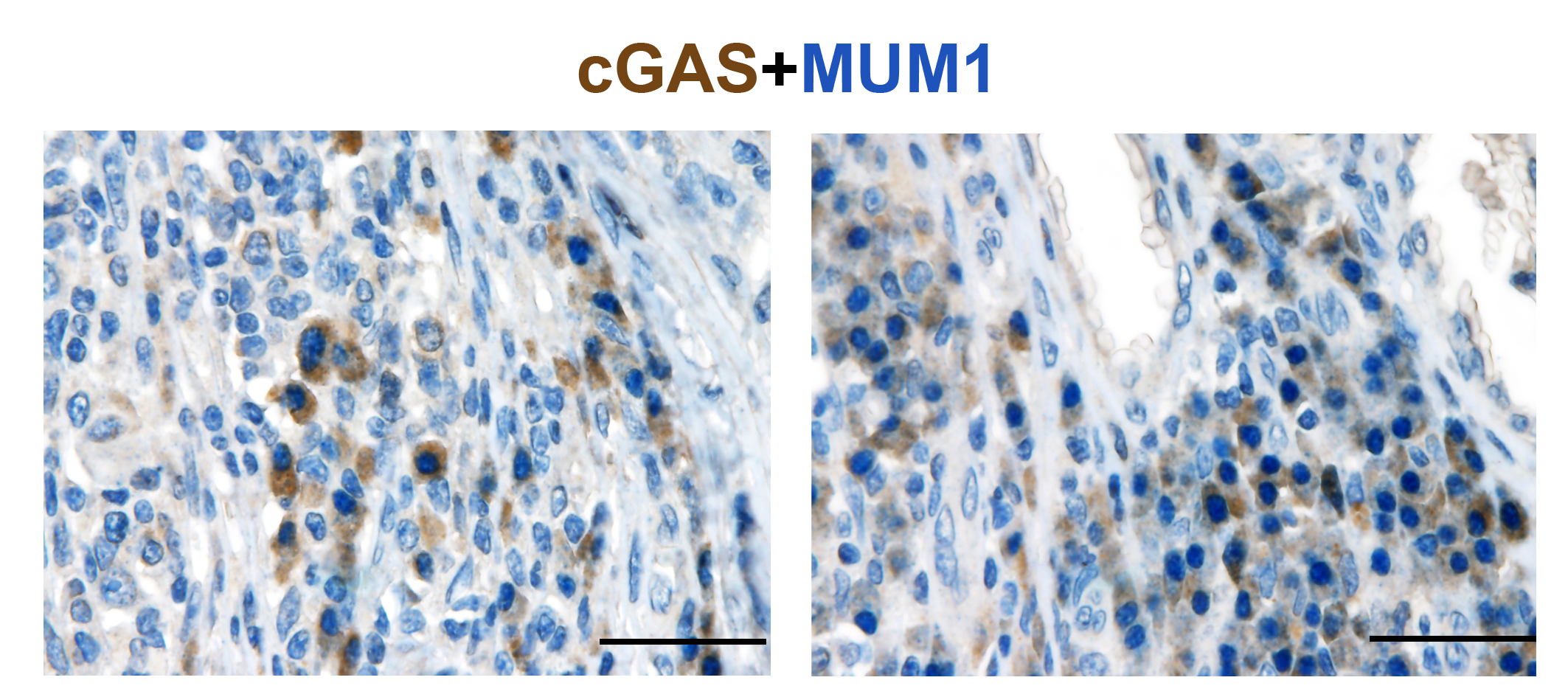

Supplement: Supplementary file 1 [file DataSheet_1.zip › Supplementary Image 1.JPEG]

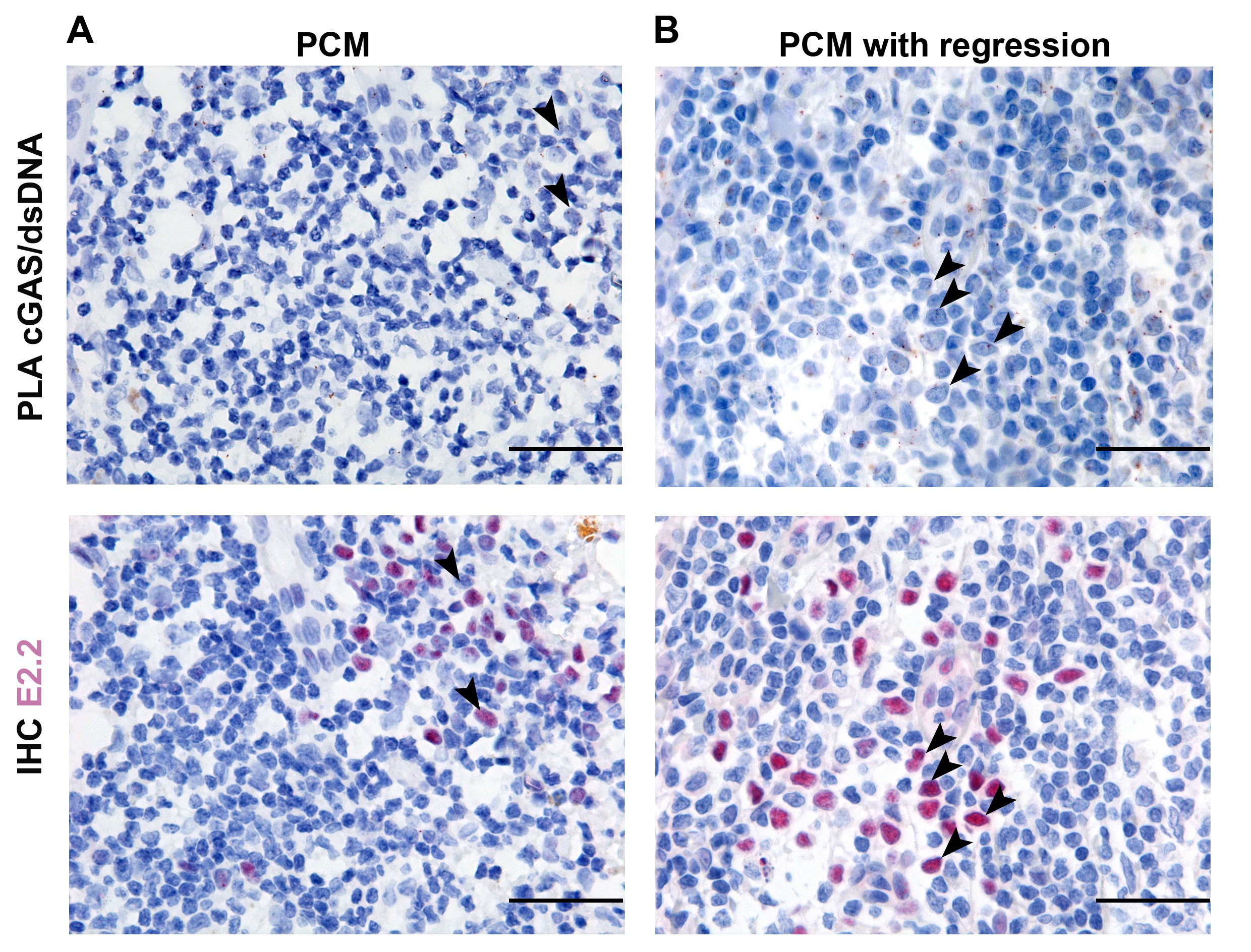

Supplement: Supplementary file 1 [file DataSheet_1.zip › Supplementary Image 2.JPEG]

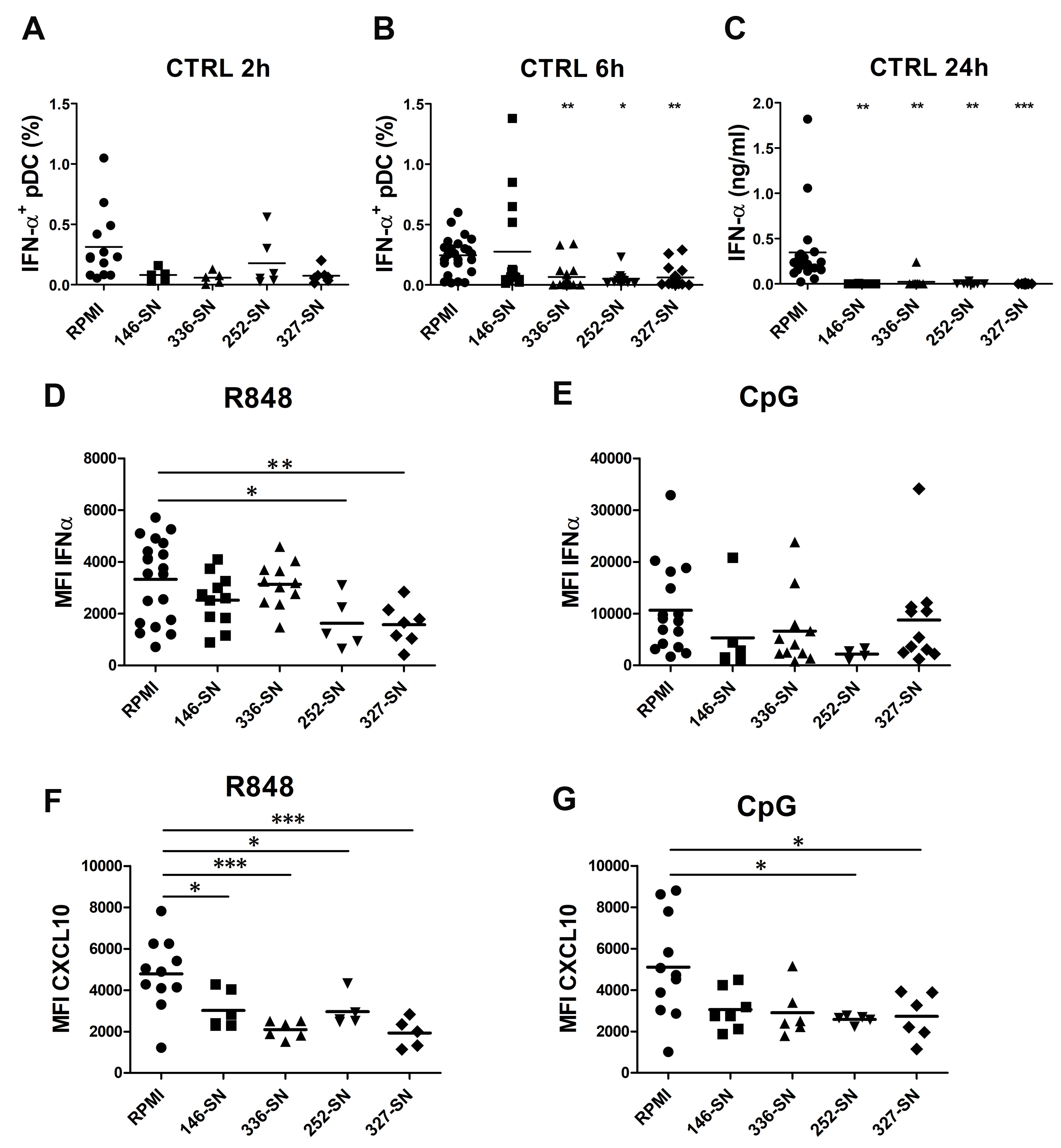

Supplement: Supplementary file 1 [file DataSheet_1.zip › Supplementary Image 3.JPEG]

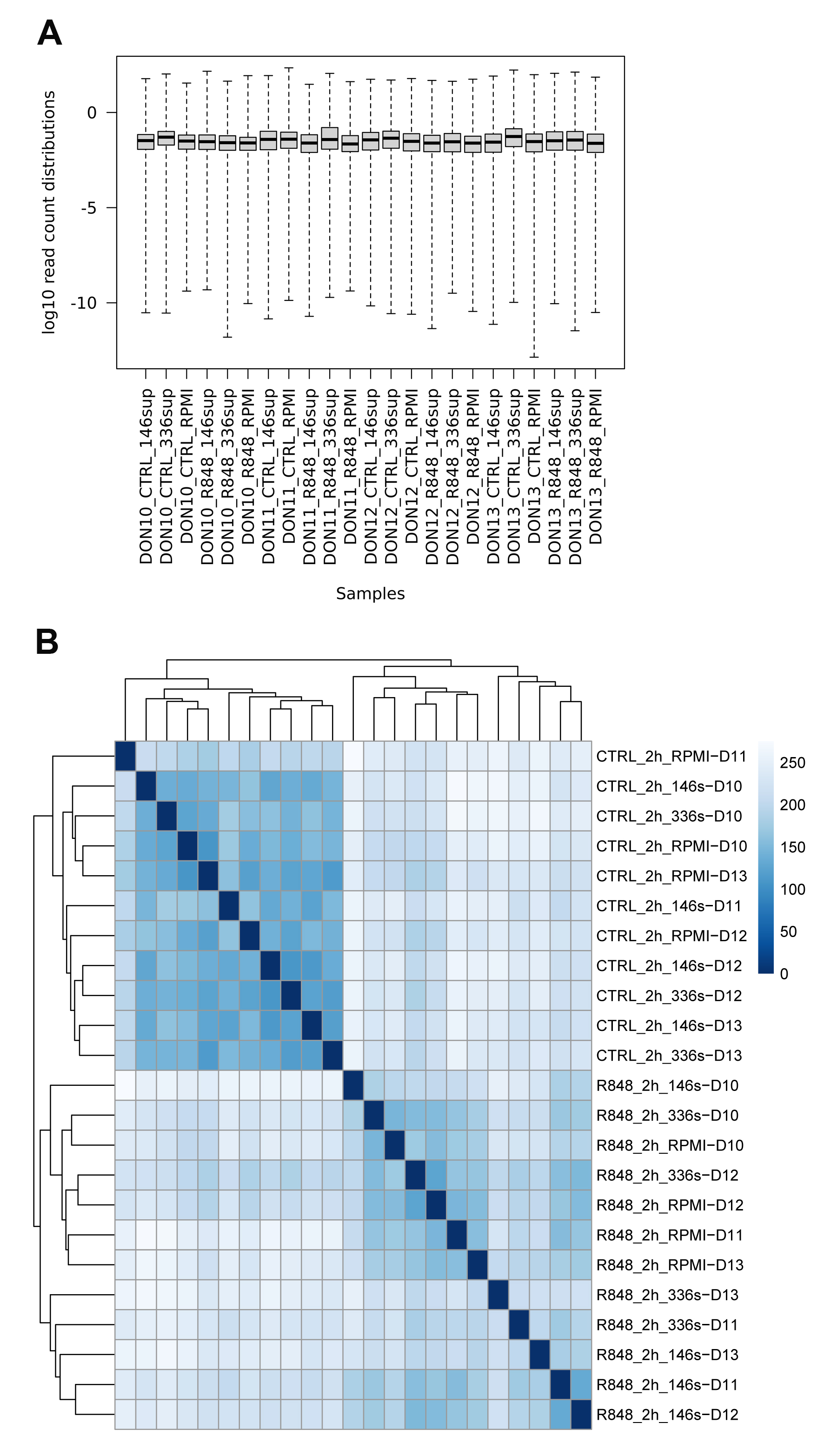

Supplement: Supplementary file 1 [file DataSheet_1.zip › Supplementary Image 4.JPEG]

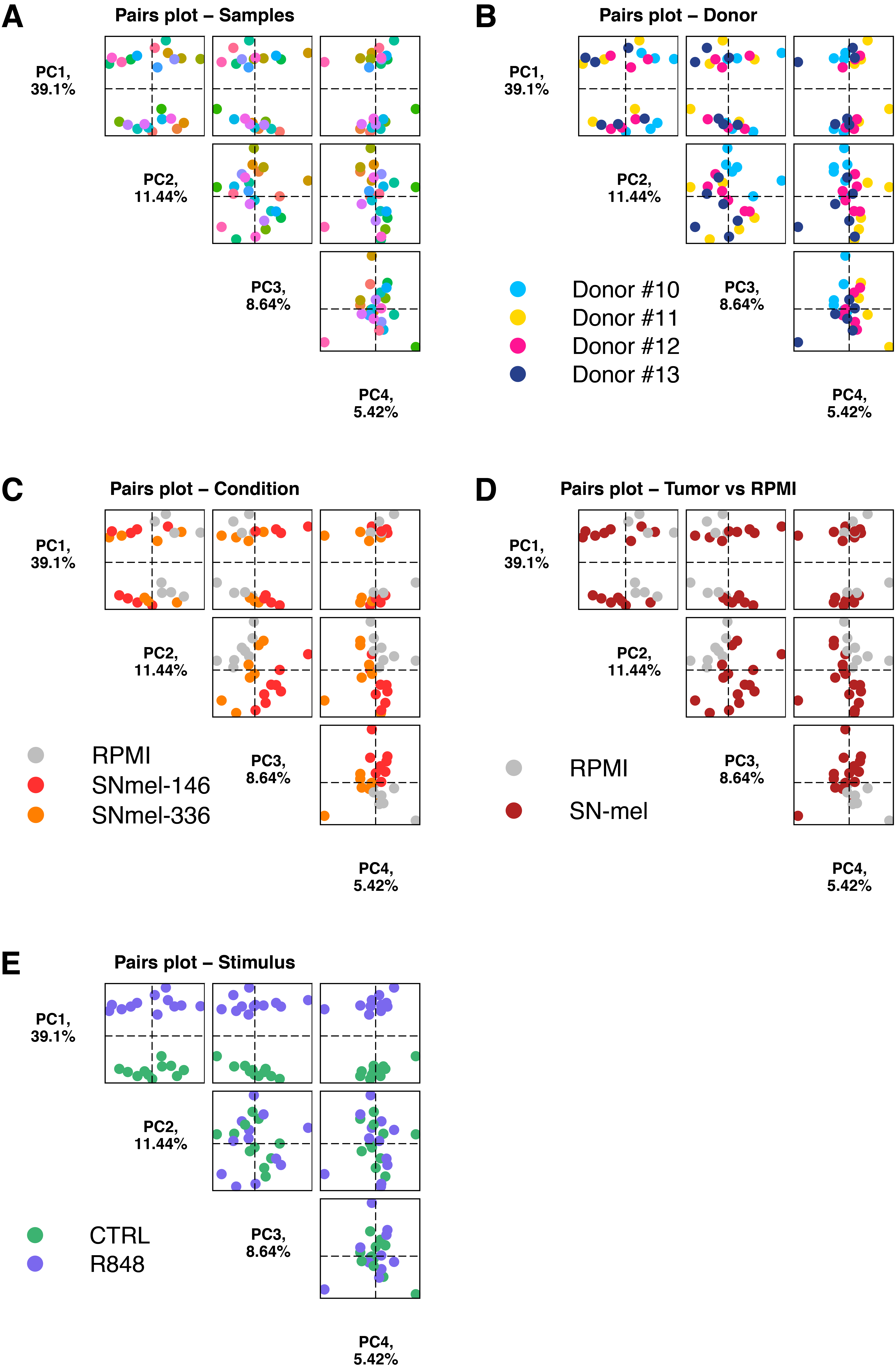

Supplement: Supplementary file 1 [file DataSheet_1.zip › Supplementary Image 5.PNG]

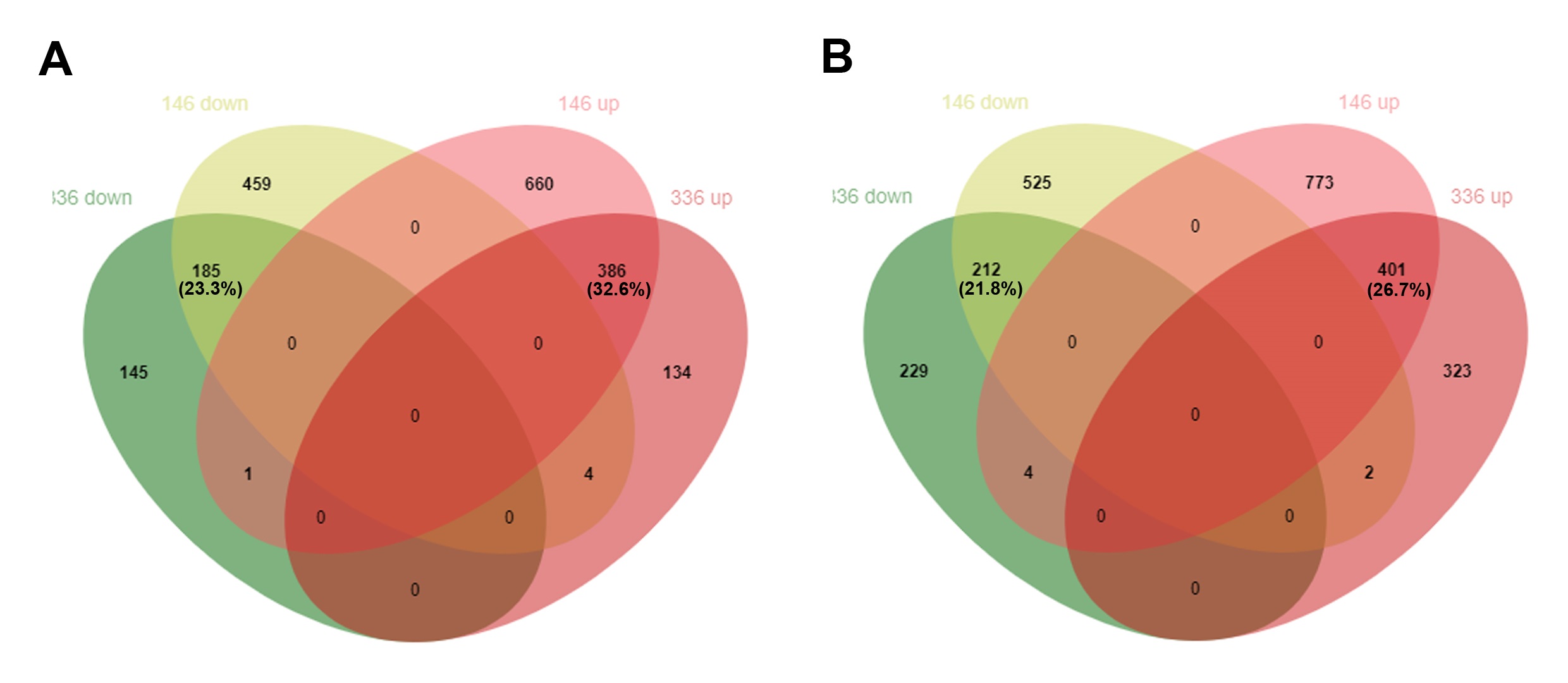

Supplement: Supplementary file 1 [file DataSheet_1.zip › Supplementary Image 7.JPEG]

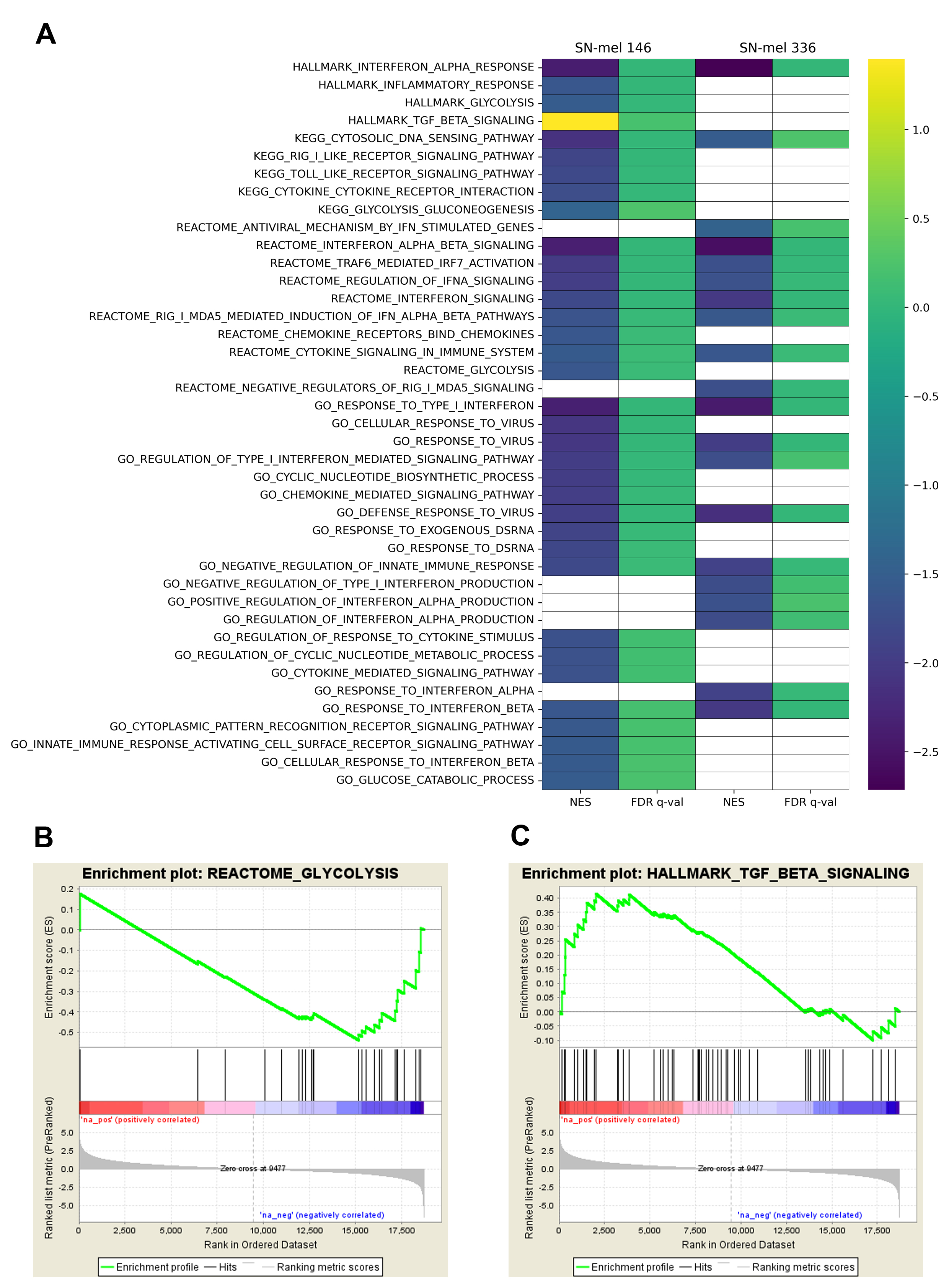

Supplement: Supplementary file 1 [file DataSheet_1.zip › Supplementary Image 8.JPEG]

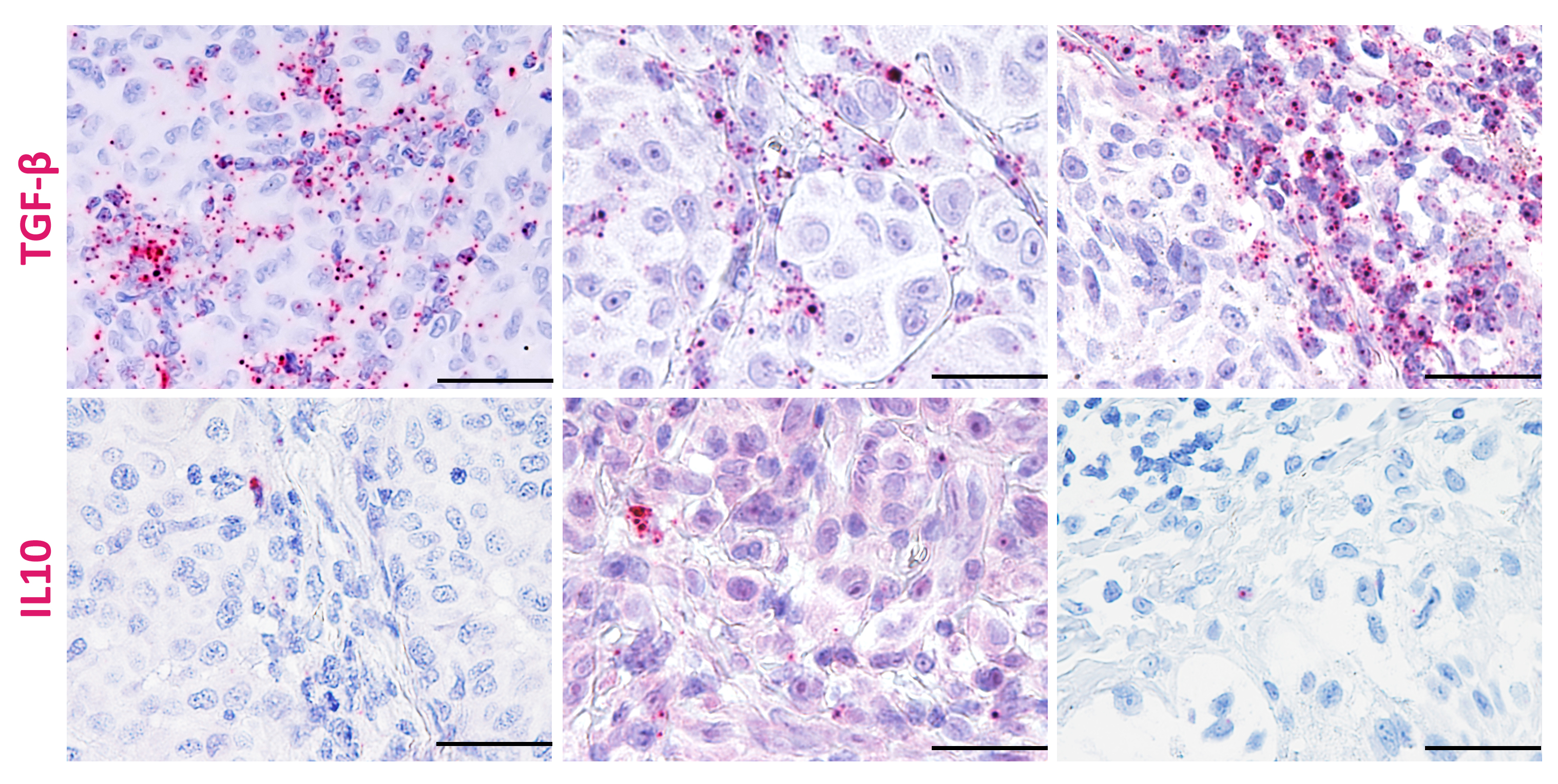

Supplement: Supplementary file 1 [file DataSheet_1.zip › Supplementary Image 9.JPEG]
